# Proline and glucose metabolic reprogramming supports vascular endothelial and medial biomass in pulmonary arterial hypertension

**DOI:** 10.1172/jci.insight.163932

**Published:** 2023-02-22

**Authors:** Bradley M. Wertheim, Rui-Sheng Wang, Christelle Guillermier, Christiane V.R. Hütter, William M. Oldham, Jörg Menche, Matthew L. Steinhauser, Bradley A. Maron

**Affiliations:** 1 Division of Pulmonary and Critical Medicine, Department of Medicine;; 2 Division of Cardiovascular Medicine, Department of Medicine;; 3 Channing Division of Network Medicine, Department of Medicine; and; 4 Division of Genetics, Brigham and Women’s Hospital and Harvard Medical School, Boston, Massachusetts, USA.; 5 Center for NanoImaging, Cambridge, Massachusetts, USA.; 6 CeMM Research Center for Molecular Medicine of the Austrian Academy of Sciences, Vienna, Austria.; 7 Department of Structural and Computational Biology, Max Perutz Labs, University of Vienna, Vienna, Austria.; 8 Vienna BioCenter PhD Program, Doctoral School of the University of Vienna and the Medical University of Vienna, Vienna, Austria.; 9 Faculty of Mathematics, University of Vienna, Vienna, Austria.; 10 Division of Cardiovascular Medicine, Department of Medicine, University of Pittsburgh Medical Center, Pittsburgh, Pennsylvania, USA.; 11 Aging Institute, University of Pittsburgh School of Medicine, Pittsburgh, Pennsylvania, USA.

**Keywords:** Cardiology, Pulmonology, Amino acid metabolism, Endothelial cells, Fibrosis

## Abstract

In pulmonary arterial hypertension (PAH), inflammation promotes a fibroproliferative pulmonary vasculopathy. Reductionist studies emphasizing single biochemical reactions suggest a shift toward glycolytic metabolism in PAH; however, key questions remain regarding the metabolic profile of specific cell types within PAH vascular lesions in vivo. We used RNA-Seq to profile the transcriptome of pulmonary artery endothelial cells (PAECs) freshly isolated from an inflammatory vascular injury model of PAH ex vivo, and these data were integrated with information from human gene ontology pathways. Network medicine was then used to map all aa and glucose pathways to the consolidated human interactome, which includes data on 233,957 physical protein-protein interactions. Glucose and proline pathways were significantly close to the human PAH disease module, suggesting that these pathways are functionally relevant to PAH pathobiology. To test this observation in vivo, we used multi-isotope imaging mass spectrometry to map and quantify utilization of glucose and proline in the PAH pulmonary vasculature at subcellular resolution. Our findings suggest that elevated glucose and proline avidity underlie increased biomass in PAECs and the media of fibrosed PAH pulmonary arterioles. Overall, these data show that anabolic utilization of glucose and proline are fundamental to the vascular pathology of PAH.

## Graphical abstract

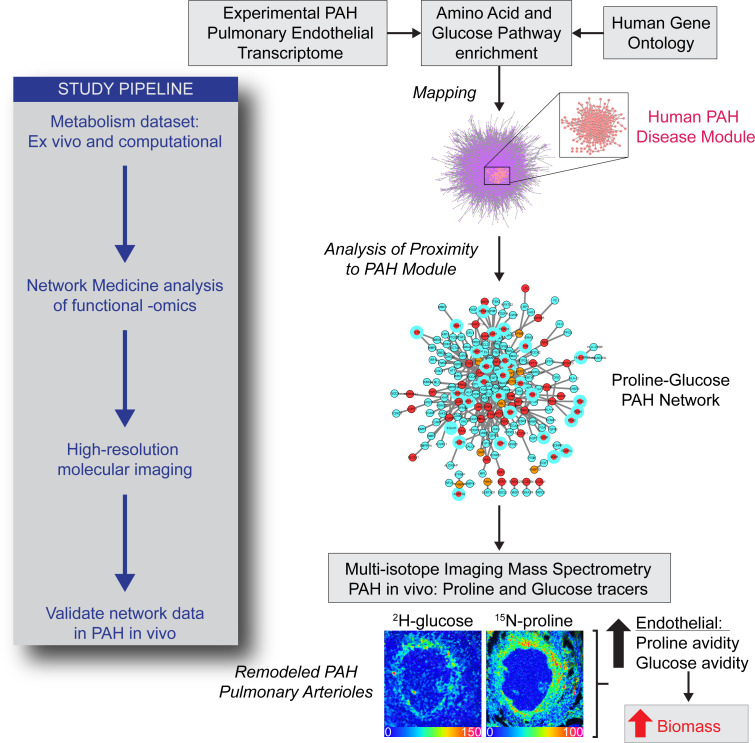

## Introduction

Pulmonary arterial hypertension (PAH) is characterized by an obstructive vasculopathy involving distal pulmonary arterioles leading to right heart failure and early mortality (1). In PAH, inflammation stimulates transcriptional responses that control metabolic reprogramming of vascular cells (2), driving fibroproliferative arterial remodeling (3). Although targeting dysregulated metabolism in PAH is feasible clinically (4), efficacy has been hampered due, in part, to cellular heterogeneity within vascular lesions and limited information on the role of metabolism to the PAH pathophenotype. Indeed, the metabolic hypothesis of PAH emerged from reductionist observations in tumor cells implicating an inherent shift toward glycolytic metabolism that is linked to changes in cell phenotype (5). By contrast, unbiased and integrated analytic strategies emphasizing functionally important transcriptomic pathways to predict the metabolic profile of vascular cells in vivo are well positioned to advance understanding of PAH pathobiology, but have not been reported previously.

A central goal of molecular imaging is to provide functional metabolic information in vivo. The most established methods involve radiotracers, such as fluoro-deoxy-glucose (FDG), coupled with PET scanning. Indeed, studies have shown increased FDG uptake in the lungs of patients with PAH and experimental disease models in vivo (6–9). However, imaging the lung at tissue scale resolution available with PET cannot characterize the cellular basis for FDG avidity, and thus, FDG-PET fails to capture cell-specific metabolic information relevant to PAH vascular pathobiology. Multi-isotope imaging mass spectrometry (MIMS) is a new imaging modality that merges in vivo stable isotope tracer methodology with nanoscale secondary ion mass spectrometry (NanoSIMS) (10).

Changes in stable isotopic labeling as detected with MIMS indicate differential metabolic activity — which we refer to as “reprogramming” — but do not reveal granular details about the identity of downstream metabolic pathways in the same manner as mass spectrometry–based flux analyses of cultured cells or tissues. Instead, the power of MIMS is attributable to the quantification of stable isotope tracers in subcellular domains at near electron microscopy resolution. Hence, MIMS provides metabolic information that is conceptually similar to PET, but with the advantage of using the full array of nontoxic stable isotope tracers (11), with the possibility of multiplexing up to 4 tracers (12), and at a resolution that localizes tracer signals to individual cells and subcellular structures (10, 13). MIMS has been used to address diverse biological questions concerning DNA synthesis, cell turnover, and aa and fatty acid metabolism in model organisms and humans (10, 14–18). MIMS has not previously been used to study the pulmonary vasculature or PAH.

In this study, we performed transcriptomic analyses of pulmonary artery endothelial cells (PAECs) isolated from an inflammatory vascular injury model of PAH ex vivo without cell culture. These data were combined with human gene ontology information to serve as the basis of a potentially novel network medicine approach that profiled the proximity of all aa and glucose pathways to the PAH disease module within the consolidated human interactome, which is a robust and validated network that includes information on 233,957 physical protein-protein interactions (19, 20). From this analysis, glucose and proline pathways were significantly close in proximity to the PAH disease module in silico. We then developed a MIMS approach to test this observation by generating multiplexed high-resolution maps of proline and glucose utilization in the PAH pulmonary vasculature in vivo. The MIMS findings validated our network predictions and indicated that reprogramming of glucose and proline metabolism underpin increased biomass in PAECs and the media of fibrosed PAH pulmonary arterioles.

## Results

### The pulmonary endothelial transcriptomic profile of inflammatory PAH.

PAECs from rats were isolated as reported previously by immunomagnetic bead selection without the use of cell culture (21). PAEC identity was determined by fluorescence-activated cell sorting gated for CD31 + Griffonia simplicifolia isolectin-B_4_ (GS-IB_4_) double positivity (Supplemental Figure 1; supplemental material available online with this article; https://doi.org/10.1172/jci.insight.163932DS1). Anti-CD31 and anti-vWF double immunofluorescence was used to confirm that the pulmonary endothelium was intact in control and PAH pulmonary arterioles in situ (Supplemental Figure 2), which is described in greater detail in the Supplemental Methods and Supplemental Table 1.

**Figure 1:**
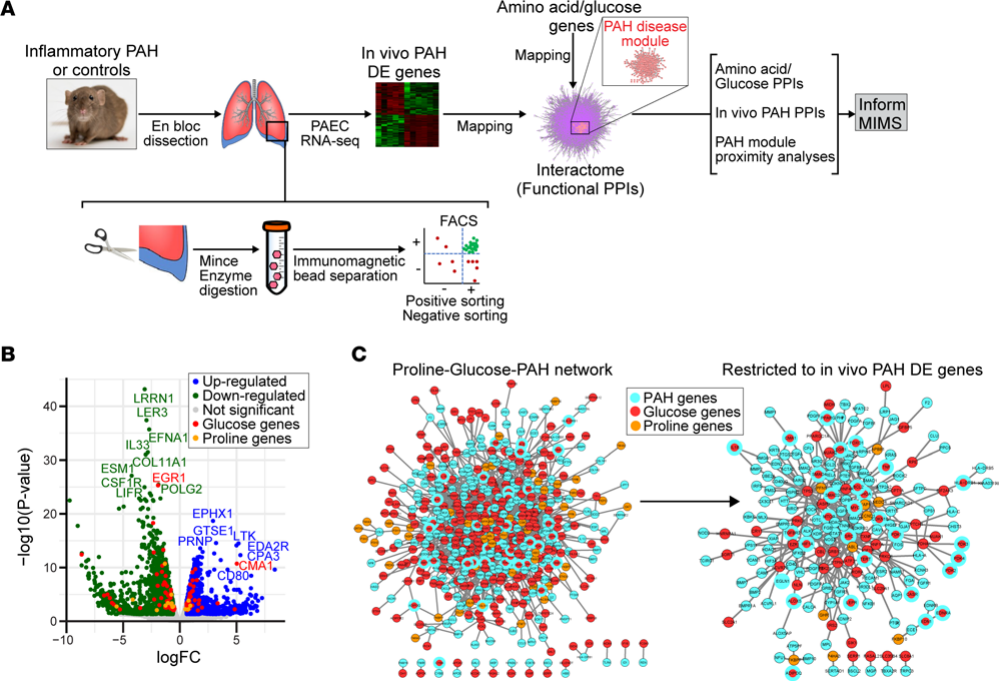
Network medicine predicts pulmonary endothelial proline and glucose pathways are functionally important in PAH. (**A**) Design and experimental throughput for the current project. PAECs were isolated from control and inflammatory PAH rat lungs, and differentially expressed (DE) genes between these groups were mapped to the consolidated human interactome, which contains information on more than 230,000 physical protein-protein interactions (PPIs). The GO database was also used to map genes associated with each aa and glucose pathway. The derivative outputs inform biological experiments focusing on the pulmonary endothelial proline and glucose programs in PAH using MIMS. SD, Sprague-Dawley. (**B**) Pulmonary endothelial DE genes between control and inflammatory PAH are presented by volcano plot. The genomic features that were up- and downregulated significantly (FDR < 0.05, *P* < 0.05; *P* values were obtained using the exact binomial test executed in EdgeR) were mapped to the consolidated interactome to identify functionally important, pulmonary endothelial PPIs in PAH. (**C**) The proline/glucose-PAH bipartite network and the proline/glucose-PAH bipartite network restricted to include only proline/glucose genes that were DE in PAH in vivo. See Supplemental Figure 5 for expanded networks from **C**.

From a total of 25,808 unique pulmonary endothelial genomic features captured by RNA-Seq analysis (i.e., FDR < 0.05, inclusive of coding and noncoding genetic elements), we identified 3,857 (14.9%) that were differentially expressed between control (*N* = 6) and inflammatory (monocrotaline) PAH rats (*N* = 6) (*P* < 1.0 × 10^–4^), of which we identified human orthologs for 2,626 (68.0%) protein-coding genes (Figure 1, A and B; and Supplemental Figure 3A). (These data are referred to hereafter as differentially expressed genes in PAH in vivo). Gene set enrichment analysis confirmed upregulation of inflammatory as well as numerous other signaling pathways with established relevance to human PAH (reviewed in ref. 22) (Supplemental Figure 3B). Human orthologs of differentially expressed genes in PAH in vivo were then mapped to the consolidated human interactome (19, 23, 24), resulting in a network of important physical protein-protein interactions that included 1,836 proteins and 6,748 interactions (Supplemental Figure 3C). We then compared this pulmonary endothelial cell PAH network with the previously described human PAH disease module inclusive of 357 genes (19, 23, 24) and found a highly significant overlap between these 2 networks (*P* < 1.04 × 10^–9^) (Supplemental Figure 3D) as well as between the pulmonary endothelial in inflammatory PAH with other inflammatory (positive control) diseases in the human interactome (Supplemental Table 2). These observations are consistent with our observation that findings using the inflammatory PAH model (25) advance a novel therapeutic approach for patients with PAH (26) and support the translational relevance of the current study.

### The association between aa-specific pathways and PAH.

We pursued a holistic approach to profiling the relevance of metabolism in PAH by focusing on all aa pathways, since protein synthesis is a key driver of fibroproliferative vascular remodeling in PAH (1). Given the previously established contributions of glucose metabolism to PAH pathobiology, we included glucose as a positive control and to identify its potential interactions with other key anabolic pathways. First, we calculated the network proximity (by mean shortest path length) to the PAH disease module for Gene Ontology (GO) gene sets organized by individual aa and glucose pathway (Table 1). Second, pulmonary endothelial genes that were also differentially expressed in PAH in vivo were organized (using GO annotation) by individual aa and glucose pathways (Table 2).

We reasoned that determining the proximity of metabolites to the PAH module in the human interactome when using both the human GO and differentially expressed genes in PAH in vivo data sets together would inform the pathways most relevant to human PAH, thereby increasing experimental rigor. We correlated the proximity to the human PAH module for GO genes versus differentially expressed genes in PAH in vivo stratified for each aa and glucose (*r* = 0.66, *P* = 0.015). To find pathways with the highest agreement between the GO versus PAH in vivo data sets and that were close in proximity to the PAH module, recursive removal of individual aa (or glucose) was performed and changes in the Pearson correlation coefficients were plotted as an “elbow” curve. In this analysis, removal of a weakly correlated aa or glucose data point improves the overall regression more than removal of a strongly correlated point. From this method, we identified 8 pathways with high agreement between the human GO and PAH in vivo data for network proximity to the PAH disease module: leucine, proline, lysine, glutamine, cysteine, arginine, asparagine, and glucose (Supplemental Figure 4A).

This analysis directed our focus to several candidate pathways for further investigation. We prioritized the proline pathway for further study based on the importance of proline to collagen (27, 28), our prior findings showing that PAECs regulate vascular fibrosis in PAH (19), and the relevance of vascular fibrosis to vascular resistance and PAH outcome clinically (29). To assess the sensitivity and specificity of network proximity findings involving proline, we tested 4 established fibrotic pathophenotypes from the literature: hypertrophic cardiomyopathy (30), idiopathic pulmonary fibrosis (31), keloid (32), and systemic sclerosis (33). Proline genes from the GO database as well as the subset of proline genes that were differentially expressed in PAH in vivo were significantly closer in network proximity to each of these respective disease modules than random expectation (Table 3 and Supplemental Table 3). By contrast, proline pathways were not significantly close to the disease modules for hyperhomocysteinemia, erythema nodosum, and rubella, which are pathophenotypes mediated by abnormalities in methyl transferases, the type IV immune response, and autoimmune cross-reactivity against gland organs, respectively, and are used here as negative disease controls (Table 3). The proximity between proline pathways, PAH, and other disease modules within the interactome is visualized in 3D in narrated Supplemental Video 1.

### Proline and glucose pathways are significantly close to the PAH disease module.

Since glucose is the dominant energy source fueling endothelial cells (34), we next aimed to determine if proline pathways with functional importance to PAH could also involve glucose pathways. We observed that glucose pathway genes were significantly close in network proximity to the human PAH module for both the GO data set (network proximity, 1.39; *P* = 1.3 × 10^–14^) and the data set from differentially expressed gene in PAH in vivo (network proximity, 1.23; *P* = 1.6 × 10^–9^). Bipartite protein-protein interaction networks showing the interactions between proline, glucose, and PAH are provided in Figure 1C (expanded views in Supplemental Figure 5), and these collective data are visualized in 3D in narrated Supplemental Video 2. These data identify the proline nodes YAP1, ABL1, PFN1, FKBP7, and CCND1 as differentially expressed in PAH in vivo and connected to a glucose node in the human PAH module (glucose nodes are listed in Supplemental Figure 4B). Further, the number of interactions involving a differentially expressed proline gene in PAH in vivo with a glucose gene in the PAH module was significantly greater compared with interactions involving a differentially expressed proline gene in PAH in vivo with a random PAH module gene (13 vs. 5.4 ± 2.8 interactions; *P* = 0.0033). These collective data suggest a distinct and functionally relevant relationship among proline and glucose metabolic pathways in PAH pathobiology.

### Development of MIMS imaging of ^15^N-proline metabolism in PAH vessels.

Our network analyses demonstrated proximity between proline and glucose pathways and the PAH disease module. When coupled with the critical importance of proline as substrate for collagen biosynthesis, these analyses provided a rationale to examine proline metabolism in vivo with MIMS for the purpose of testing 2 a priori predictions: (1) PAECs in inflammatory PAH demonstrate evidence of reprogramming of proline metabolism, and (2) the network proximity of glucose and proline pathways translates into colocalization of glucose and proline incorporation into the remodeling pulmonary arterial wall.

**Figure 2:**
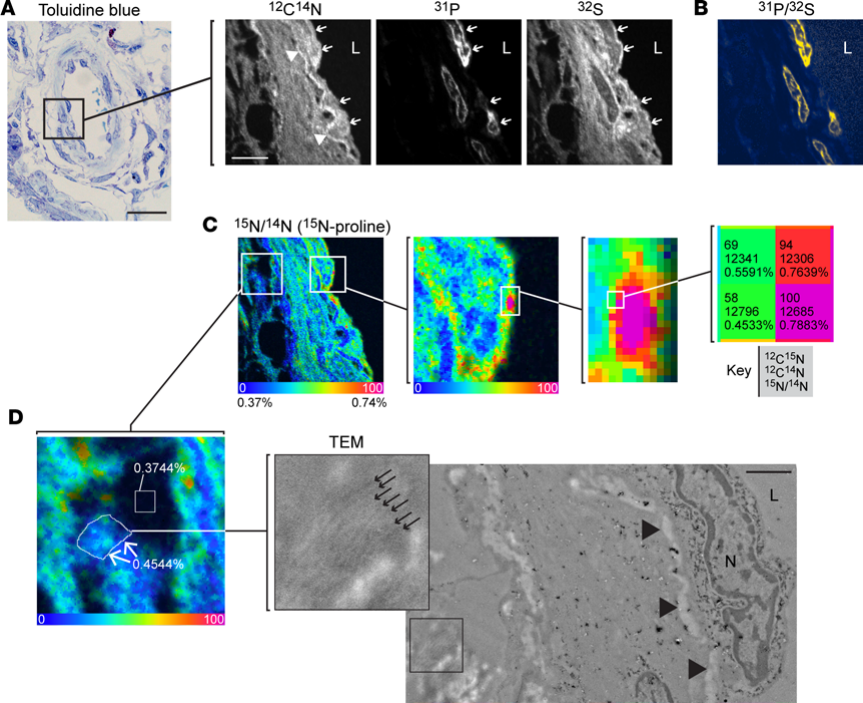
Quantitative mapping of proline utilization at high spatial resolution in PAH vessels. (**A**) Representative pulmonary artery from the inflammatory PAH model in a resin-embedded section stained with toluidine blue. This section is adjacent to a section mounted on a silicon wafer for MIMS imaging (right). Single ion images provide histological details and stereotypical vascular features. The ^12^C^14^N image reveals tissue boundaries including the endothelial-lumen interface. Elastin appears hyperintense (white). The ^31^P image identifies nuclei due to the phosphorus content of chromatin. ^32^S images resemble ^12^C^14^N images, but the nuclei appear dark. (**B**) As such, the ratio of ^31^P to ^32^S yields particularly pronounced nuclei. Endothelial cells were identifiable by their flattened appearance and direct interface with the lumen (arrows). (**C**) Hue saturation intensity images display the isotope ratio measurements and map incorporation of ^15^N-proline. The lower end of the scale (blue) is set to the background ratio (0.37%) and expressed as a percentage above background (represented by 0). The upper end of the scale is set to reveal differences in labeling (0.75% = 100% above background). (**D**) Correlative imaging of adjacent thin section by TEM. Inset shows collagen fibers (small arrows). Large arrowheads: elastin; N, nucleus; L, lumen. Region of extracellular matrix with abundant collagen fibers is labeled with ^15^N-proline (0.4544%). The indicated square region where there is no tissue (resin) is approximately at natural abundance (0.3744%). Scale bar: 20 μm (toluidine blue), 5 μm (MIMS, **A**–**C**).

We first developed a new ^15^N-proline MIMS imaging protocol, as MIMS had not been used to study the lung or to study proline metabolism to our knowledge. Therefore, we first established a MIMS approach to map proline utilization at high spatial resolution in the pulmonary vasculature and include here a synopsis of our potentially novel methodology. Our proline labeling protocol reflected prior in vivo experience with other aa tracers, such as ^15^N-glutamine (15). We performed initial analysis of lung sections using approaches that we have applied to the heart and systemic vasculature (35–37). We imaged lung sections in “chain analysis” mode where sequential adjacent square fields (typically 40–60 μm in diameter) are captured and stitched together. This initial analysis demonstrated stereotypical lung histological features and confirmed detectable ^15^N-proline labeling (Supplemental Figure 6); however, the yield of target vessels was low relative to the high analytical time required because the more prevalent airways and alveoli result in analytical dead space. To address this throughput challenge, we imaged lung sections with differential interference contrast (DIC) microscopy to identify vessels prior to MIMS analysis. Pulmonary vessels were identifiable in the resin-embedded sections mounted on silicon wafers. We then incorporated a DIC-guided selection of vessels into our analytical protocol (Supplemental Figure 7), such that each imaging field captured a vessel or a substantial fraction of a vessel. A vessel wall from a PAH rat labeled with ^15^N-proline is shown in Figure 2A, in which ^12^C^14^N, ^31^P, and ^32^S mass images demonstrate stereotypical and complementary histological details. Variability in ^12^C^14^N intensity demonstrates cellular and tissue morphology. Areas without cellular or extracellular tissue material appear black. In contrast, certain tissue features appear hyperintense, such as undulating elastin bands. ^31^P images identify nuclei analogous to fluorescent DNA stain due to the high phosphorus content of chromatin (10, 16). ^32^S images resemble ^12^C^14^N images with notable exceptions, including high signal from intracellular granules found in granulocytes and low signal in nuclei (10, 16). As such, images representing the ^31^P over ^32^S ratio further distinguish nuclei (Figure 2B).

These mass images guided selection of regions of interest (ROI) corresponding to cellular constituents of the pulmonary arteries: RBCs based on stereotypical features and intraluminal location; endothelial cells by an elongated appearance and direct interface with the lumen; and medial cells designated as those located deep in the endothelium. We visualized the isotopic ratio as a hue saturation intensity (HSI) transformation in which the lower bound of the scale (blue) is set to the natural background abundance and the high end of the scale is set to elucidate regions where the isotope ratio is above the background indicative of tracer incorporation (Figure 2C). Our initial examination confirmed effective labeling of the vessel wall in PAH rat lungs with obvious heterogeneity in signal intensity, inclusive of small punctate areas of intense labeling (hotspots: Figure 2C). In contrast, the elastin appeared relatively unlabeled. To assess regions of labeling in a quantitative and unbiased fashion, we leveraged a feature in the OpenMIMS software to identify the most intense hotspots (5 × 5 pixels, *N* = 200) and the least intense spots (5 × 5 pixels, *N* = 200) (Supplemental Figure 8). The ^15^N-proline hotpots were highly concentrated in extranuclear regions of the pulmonary endothelium, whereas ^15^N-poor spots were concentrated in the elastin extracellular matrix consistent with the visual impression. We next performed correlative imaging with transmission electron microscopy (TEM) (Figure 2D). Even though the standard MIMS samples are not prepared with traditional TEM contrast agents, we were able to visualize many of the same structural features with TEM, including nuclei and elastin bands. Extracellular regions, enriched with collagen fibers, were also evident in the TEM image, where we detected ^15^N labeling.

These data establish a ^15^N-proline labeling and MIMS protocol to quantitatively measure proline metabolism at subcellular resolution. With MIMS, we tracked ^15^N-proline into remodeling lung arteries in the inflammatory PAH model and observed heterogeneous incorporation in the vessel wall, including the collagen-rich extracellular matrix.

### Reprogramming of proline and glucose metabolism in endothelial cells.

**Figure 3:**
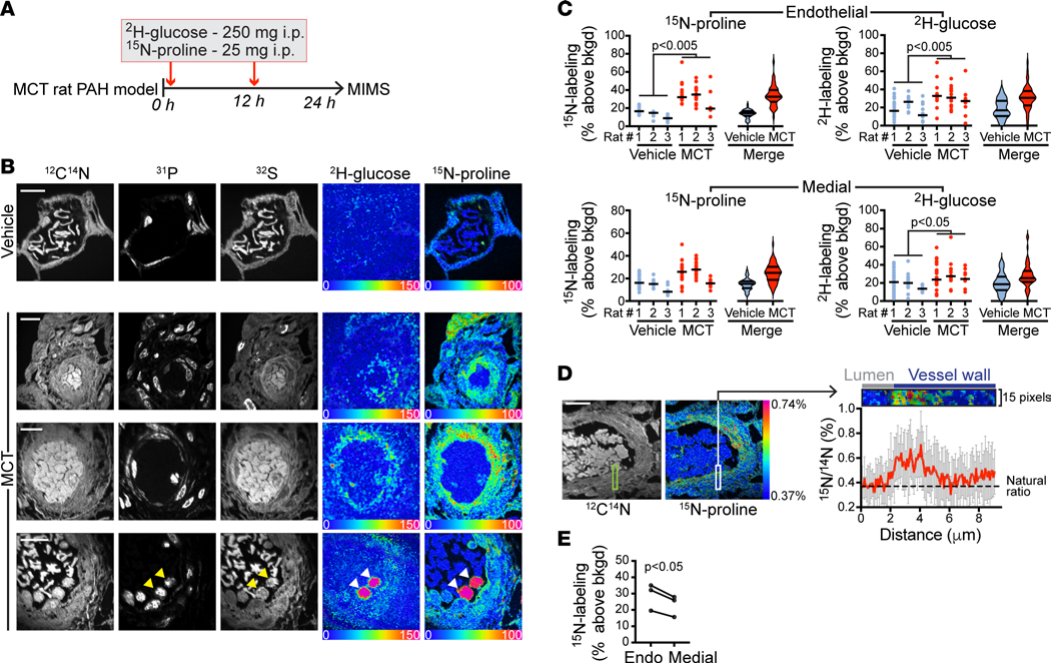
Reprogramming of proline and glucose metabolism in remodeled pulmonary arteries. (**A**) Stable isotope labeling protocol. MCT, monocrotaline. (**B**) MIMS images of pulmonary vessel from control rat (top row) and inflammatory PAH vessels (bottom 3 rows). Stereotypical features of remodeling vessels are evident in ^12^C^14^N, ^31^P, and ^32^S images, including thickening of the walls and increased cellularity. HSI images demonstrate increased ^2^H-glucose (^2^H/^1^H image) and ^15^N proline labeling (^15^N/^14^N image) in the walls of remodeling vessels. Bottom row, arrowheads indicate 2 intensely labeled nucleated WBCs in the lumen. Scale bars: 10 μm. (**C**) Dot plots of endothelial (top) and medial cell (bottom) proline (left) and glucose (right) labeling in inflammatory PAH vessels versus control vessels. Data in violin plots are presented as median, IQR. Each dot represents a nucleated cell. *P* values calculated by the nested ANOVA method. (**D**) The ^15^N/^14^N ratio for pixels of the indicated region of the vessel wall mapped as a function of distance from the origin in the lumen (top). (**E**) Median endothelial cell labeling relative to median medial cell labeling (*n* = 3 PAH rats; *P* values calculated by the Student’s paired 2-tailed *t* test). Representative images provided in each instance.

To test for metabolic alterations in PAH pulmonary endothelial cells in vivo, we analyzed PAH vessels relative to control (vehicle) rats after administration of a cocktail containing both ^15^N-proline and ^2^H-glucose (Figure 3A). We used a ^2^H-glucose dose that was similar by weight to that we have previously used in murine studies (15). ^12^C^14^N, ^31^P, and ^32^S mass images demonstrate consistent vascular remodeling as indicated by thickening of the vessel wall and increased cellularity (Figure 3B). Isotope ratio images demonstrated augmentation of ^15^N-proline and ^2^H-glucose labeling (Figure 3B), which was confirmed by extraction of single-cell-level ratio data (Figure 3C). In a merged analysis, the ^15^N-proline labeling was increased 2.2-fold in PAH endothelial cells relative to control (PAH median 32.6%, IQR 27.0–39.9, above background vs. control median 14.4%, IQR 11.6–16.7), and the ^2^H-glucose labeling was increased 1.8-fold in PAH endothelial cells relative to control (PAH median 30.7%, IQR 22.3–38.2, above background vs. control median 17.0%, IQR 10.6–27.4) (Figure 3C). A directionally similar augmentation was observed in the medial cells as the ^15^N-proline labeling was increased 1.6-fold in PAH mural cells relative to control (PAH median 24.8%, IQR 18.7–30.1, above background versus control median 15.1%, IQR 11.4–17.3) and the ^2^H-glucose labeling was increased 1.3-fold in PAH medial cells relative to control (PAH median 25.3%, IQR 21.033.2, above background versus control median 18.5%, IQR 12.1–26.9) (Figure 3C). However, the magnitude of medial labeling was not as high as in the pulmonary endothelium (Figure 3, D and E; and Supplemental Figure 9A).

We also examined RBC labeling distributions, reasoning that they would be a good control for consistency of label delivery within and between groups. RBCs demonstrated detectable proline and glucose labeling. Small subpopulations of highly labeled outliers were observed in vehicle and PAH rats at a frequency that may represent immature RBCs (38). Importantly, the similar glucose and proline labeling of RBCs across animals, including both PAH and controls, suggested uniform label delivery to the model (Supplemental Figure 9B). Collectively, these stable isotopic labeling data demonstrate metabolic changes in the pulmonary arteries in the inflammatory PAH model, which cannot be explained by technical differences in label delivery.

In ^15^N-proline ratio images, we also observed higher labeling intensity in the pulmonary endothelial layer, relative to the media. Line profiling of the ^15^N-proline signal as a function of depth in the vessel wall provided quantitative validation of this observation, as did the cell level labeling distributions: the median ^15^N labeling of endothelial cells in PAH vessels was significantly higher than corresponding medial cells in the same animals (Figure 3E). In a prior study, we examined glucose labeling with MIMS in inflammatory atherosclerotic lesions in mice (35) and in that context did not observe augmented endothelial labeling relative to medial cells (Supplemental Figure 10). Nonetheless, the degree to which the endothelial MIMS signal is specific to PAH pathology, or a more general phenomenon of inflamed tissues (Supplemental Table 2), remains an important unanswered question. Collectively, these analyses of ^15^N-proline and ^2^H-glucose tracer incorporation into pulmonary vessels in the inflammatory PAH model suggest reprogramming of glucose and proline metabolism, with marked proline avidity in the pulmonary endothelium as predicted by our network analyses.

### Anabolic convergence of glucose and proline as substrate for biomass in remodeling vessels.

**Figure 4:**
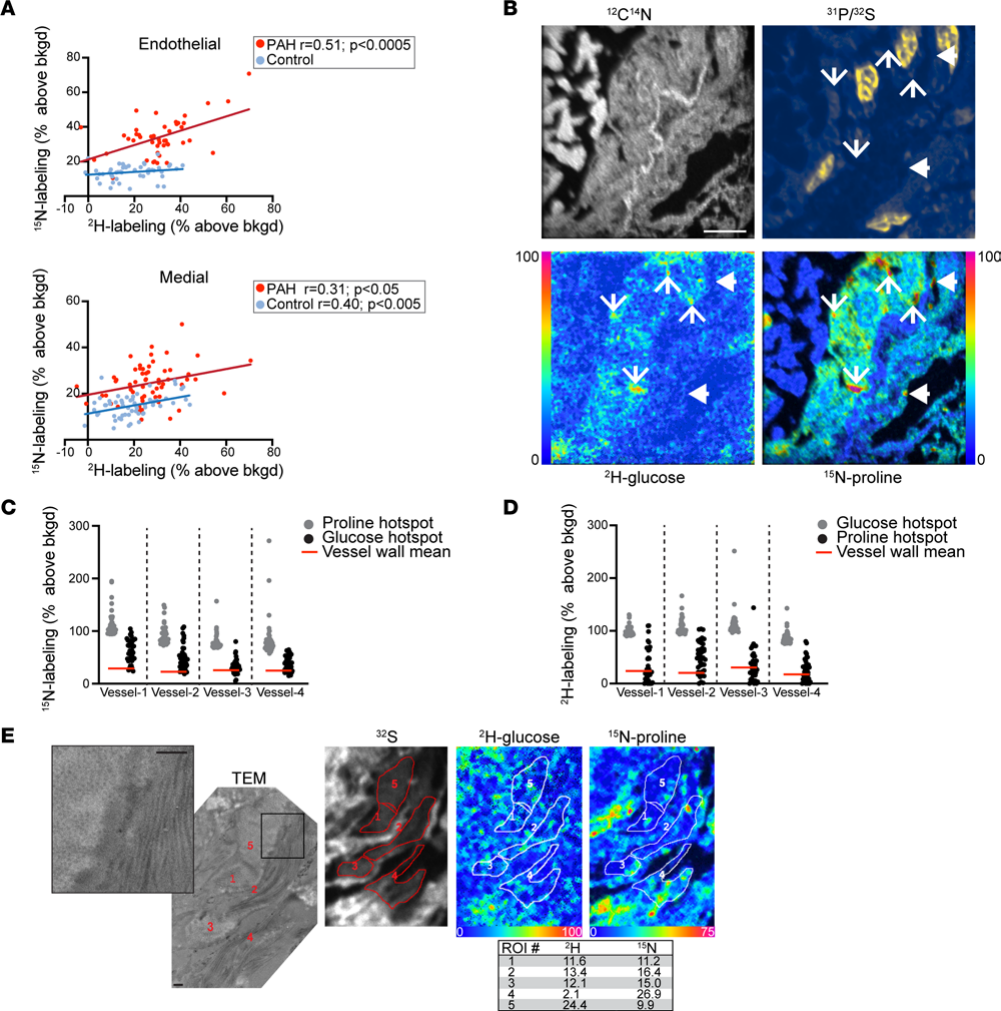
Anabolic convergence of glucose and proline as substrate for biomass in remodeling vessels. (**A**) Top: MIMS quantification of proline utilization correlates with glucose utilization in the endothelial cells of inflammatory PAH vessels, but not control vessels. Bottom: MIMS quantification of proline utilization by medial cells correlates with glucose in both PAH and control vessels. Correlations assessed by linear regression model. (**B**) High-resolution MIMS imaging demonstrates punctate hotspots of hyperutilization of glucose and proline. Line arrows, hyperintense in both glucose and proline; arrowheads, hyperintense in proline but not glucose. Scale bar: 5 μm. (**C**) ^15^N-labeling distributions for glucose hotspots (*n* = top 40), which are largely above the mean for the vessel wall (inclusive of nonhotspots and hotspots) (red line) and partially overlap with the distribution of top 40 ^15^N-proline hotspots (gray). (**D**) Complementary analysis to **C** as the ^2^H-glucose labeling for the top ^15^N-proline hotspots partially overlaps with the distribution of top 40 ^2^H-glucose hotspots. In **C** and **D**, the mean signal for each vessel wall is provided as a reference (red line). Hotspots were 5 × 5 pixels. (**E**) TEM adjacent to section imaged with MIMS. TEM image inset demonstrates extracellular matrix with collagen fibers arrayed in both cross section (left) and longitudinally (right). ROI were generated to correspond to regions of ECM on the TEM and the data extracted and expressed as a percent above background (table) indicative of glucose and proline labeling. Scale bars: 500 nm.

Having demonstrated augmentation of substrate utilization in PAH vessels, we next tested for correlation between proline and glucose labeling. This type of correlative analysis is possible, as MIMS allows for the simultaneous detection of several isotopes in the same nanovolume of sputtered sample material. We first examined this question at the cellular level, finding a strong correlation between ^2^H-glucose and ^15^N-proline labeling in endothelial cells in the inflammatory PAH vessels but not in control vessels (Figure 4A). In contrast, glucose labeling predicted proline incorporation in medial cells in both inflammatory PAH and control.

We next sought to map proline and glucose utilization at higher resolution. While the NanoSIMS instrument can achieve a lateral resolution less than 50 nm, this comes at a throughput cost; therefore, routine operation is at the 100–200 nm range of lateral resolution. We performed higher resolution imaging, however, in a subset of vessels to map label incorporation into subcellular and extracellular domains. In these analyses, we identified numerous labeling hotspots of particularly elevated ^2^H-glucose and ^15^N-proline labeling intensity. Of note, there were also puncta with preferential labeling by just 1 of the tracers (Figure 4B). We next assessed the degree to which the 2 labels associated in the vessel wall using the OpenMIMS software, focusing on the most intense hotspots (5 × 5 pixels, *n* = 40) for both ^2^H-glucose and ^15^N-proline (Figure 4, C and D). Many, but not all, of the hotspots for ^2^H-glucose label also tended to be high in ^15^N-proline and vice versa. Finally, we also performed correlative TEM imaging and found ^2^H-glucose and ^15^N-proline labeling of collagen-rich extracellular areas in the vessel wall (Figure 4E). (The correlative histological pattern of pulmonary arterial fibrosis in the PAH model is provided in Supplemental Figure 11.)

Importantly, the labeling pattern of these putative fibrotic regions was heterogeneous. Labeling hotspots suggest areas in which intense biosynthetic action occurred during the 24-hour labeling period preceding rat sacrifice. By contrast, domains with minimal labeling are consistent with previously remodeled regions or components characterized by slower turnover, such as the elastin fibers (Figure 2D and Supplemental Figure 8). Since MIMS ostensibly involves analysis of tissue components such as proteins and nucleic acids that are preserved by tissue fixation and dehydration (15), including extracellular matrix (Figure 4E), these data collectively suggest convergence of glucose and proline metabolism to support generation of biomass in the remodeling vessel. Moreover, the heterogeneity of glucose and proline labeling, which was appreciable at the cellular (Figure 3 and Supplemental Figure 12) and subcellular levels (Figure 4 and Supplemental Figure 12), indicates nanoscale differences in anabolic utilization of glucose and proline that would not be appreciable with tissue-scale methods, such as FDG-PET.

Our MIMS data suggest that PAECs are highly avid for both glucose and proline. These data were supported by findings in a separate cohort of control and inflammatory-PAH rats in which right ventricular systolic pressure (RVSP) assessed by cardiac catheterization correlated positively with mRNA expression quantity of the proline transporter SLC38A1 (*r* = 0.7, *P* = 0.021) and glucose transporter SLC2A1 (*r* = 0.75, *P* = 0.01). The expression profile of SLC38A1 and SLC2A1 were strongly correlated (*r* = 0.94, *P* < 2.2 × 10^–16^) as well (Supplemental Figure 13). Overall, these findings are internally consistent with our network medicine and MIMS data suggesting activation of glucose and proline regulatory pathways in inflammatory PAH.

## Discussion

We implemented what we believe to be a novel network medicine methodology to study metabolism in PAH, initiated through the analysis of functionally relevant metabolic pathways for all aa and glucose using pulmonary endothelial transcriptomic data from an inflammatory model of PAH and computational data from humans. These data predicted that proline and glucose pathways are interrelated and important in human fibrotic pathophenotypes, including PAH. However, we recognized the limitations of inferring metabolic function from computational strategies alone. To validate these computational findings, we developed a MIMS imaging approach that revealed augmentation of glucose and proline utilization in inflammatory PAH vessels, including the pulmonary endothelium. Collectively, findings from this study suggest that plasma proline and glucose substrates converge to support biomass generation in remodeling pulmonary arterioles, thereby expanding the metabolic basis of PAH.

Prior studies on metabolism in pulmonary vascular disease have emphasized the Warburg phenomenon (5), interrogated single biochemical reactions linked to mitochondrial or monogenic abnormalities (39, 40), or utilized multiplex platforms to identify metabolomic signatures that characterize patient subgroups (41, 42). Here, we used network medicine to integrate various big data elements (i.e., metabolic pathways, transcriptomics, and protein-protein interactions) for experimental validation. In doing so, this work achieves a scientific benchmark (43) in which interconnecting omics optimize the specificity and rigor of outputs (44, 45). Since an overarching objective of this study was to ignore a priori assumptions regarding potential links between specific aa and PAH, a strategy such as network medicine that could reduce the initial data set according to functionally relevant pathways was essential. Nevertheless, this work is, to our knowledge, the first to show that interfacing gene ontology with transcriptomic data acquired ex vivo as a collective — and analyzed further by calculating the proximity of these outputs to a human disease module — is effective for generating potentially novel and testable discoveries of direct relevance to human disease.

Like cancer, PAH can be viewed as a state of pathological biomass generation, because thickening of the distal pulmonary arterial wall is dramatic and driven by a combination of cellular proliferation and extracellular matrix deposition. Augmented proline incorporation into the vessel wall is in line with this paradigm, since (i) expansion of biomass cannot occur without protein synthesis and (ii) proline itself is a critical aa substrate for collagen biosynthesis and fibrosis (28). By mapping proline incorporation into the remodeling vessel at high spatial resolution with MIMS, however, we found marked heterogeneity in the regions of ^15^N-proline labeling, including significantly higher proline utilization in the pulmonary endothelial layer. We have demonstrated previously the role of the endothelium in collagen biosynthesis (19, 46); therefore, these data provide in vivo functional support that the endothelium is particularly susceptible to metabolic reprogramming, even relative to mural cells, and overall is an active player in biomass generation in remodeling vessels.

Prior to our study, the most direct, functional evidence of metabolic reprogramming in PAH in vivo involved FDG-PET demonstration of augmented tracer uptake in the lungs of experimental animal models and human patients (6–9). There are 4 key methodological aspects of MIMS that are distinct from PET and contextualize the significance of increased glucose labeling in the wall of the remodeling pulmonary vasculature. (i) Moving from tissue scale resolution with PET down to subcellular resolution with MIMS directly localizes increased avidity for glucose to affected arteries in the pulmonary vasculature. (ii) FDG tracer tracks glucose uptake but not its downstream metabolism because the deoxygenated form of glucose cannot be used for glycolysis. In contrast, stable isotopically enriched glucose is incorporated into metabolic pathways and, therefore, tracks both uptake and downstream metabolism. Like PET, however, MIMS tracer measurements do not provide specificity of downstream metabolites. (iii) Tissues are processed ex vivo with histological methods, including aldehyde fixation and alcohol dehydration, and, therefore, MIMS ostensibly detects tracer incorporation into the fixable biomass (15). (iv) Multiplexing demonstrates colocalization of glucose labeling with putative incorporation of proline into newly synthesized protein. Therefore, our MIMS analyses validate the collective FDG-PET literature in PAH but at a resolution that provides arguably the most direct in vivo evidence of glucose reprogramming in remodeling vessels.

Intracellular glucose metabolism is arguably more complex than proline metabolism, with a greater variety of metabolic fates for glucose relative to proline. Maximal ATP production for each glucose molecule involves completion of glycolysis and oxidative phosphorylation; however, metabolites at multiple nodes in this energy generating pathway can be shunted to support components of biomass, including nucleic acid, aa, and fatty acid synthesis. It is well established that cancer cells use glucose intermediates for biomass, even when there is sufficient oxygen to support oxidative phosphorylation and maximal energy generation. Our detection of glucose incorporation into new biomass in remodeled vessels is also consistent with metabolic features commonly exhibited by tumors, thereby providing in vivo support to the granular ex vivo cellular flux analyses of PAH cells that have established the concept of glycolytic reprogramming in PAH (6, 40, 47).

Although our computational methods and prior work directed emphasis on proline, numerous other metabolic targets have been shown to be important in PAH. Some molecules, such as glutamine (48) and L-2-hydroxyglutarate (49), emerged from our computational analyses or have been identified as important in PAH previously, and were not tested directly in this study. We used validated resources to collect genes associated with aa/glucose regulatory pathways; however, it is likely that those databases, as is the case for the interactome (50) and the PAH disease module (19, 23, 24), are incomplete. In the case of the GO database, some genes are associated with functions broadly related to aa biofunctionality, such as prolyl hydroxylation in the case of proline, thereby limiting the specificity of relevant results to metabolism per se. Furthermore, the relationship between mRNA transcript quantity and protein expression is variable in some studies (51). Thus, overall, the absolute precision of our computational methods cannot be known, which reinforced the need for a unique, high-resolution imaging modality such as MIMS to validate our models. We used a well-published and valid PAH animal model to test our network medicine findings (16, 22), but alternative models could have been used, which might have generated different results.

Rat PAECs were isolated for transcriptomic analysis using a method published by our group (21) that does not require the use of cell culture or sequential cell passaging on plastic, which we pursued in this study to avoid inadvertently manipulating the PAH-pulmonary endothelial transcriptome. Although our fluorescence-activated cell sorting approach to PAEC identification was rigorous and suggested that the cell population used in this study was homogenous, the possibility that nonendothelial cells were included in bulk RNA-Seq could not be excluded, emphasizing the importance of validating our pulmonary endothelial transcriptomic and in silico findings empirically with MIMS.

One limitation of this study is that our MIMS metabolic measurements were conducted in a single inflammatory PAH model and we did not directly analyze our findings in human patients with PAH. The extensive decades-long history of using stable isotope tracers even in the most vulnerable human populations (11), coupled with our prior experience conducting first-in-human MIMS experiments (16), lays the foundation to study the pulmonary vasculature in human patients with PAH. Looking to the future, the biggest challenge with any human MIMS imaging of an internal organ like the lung is that tracer administration must be timed with surgical sampling that is part of usual clinical care. In a study of cardiomyocyte DNA synthesis in infants with congenital heart disease, for example, this was accomplished by labeling patients prior to planned surgical repair (37). Although the lung tissue of patients with PAH is not routinely sampled, we envision a translational strategy focused on studying the small subset of patients with PAH who undergo lung transplantation, where stable isotope tracers could be administered in the preoperative period with subsequent sampling of the explanted lung for MIMS analysis.

In summary, multiplexed MIMS imaging maps glucose and proline utilization at subcellular resolution in diseased pulmonary vessels in vivo and demonstrates metabolic reprogramming at a resolution not previously achievable with standard molecular imaging approaches. These observations were informed by findings from a network medicine analysis that predicted dynamic and inter-related modulation of glucose and proline pathways in PAH. Our MIMS measurements also provide quantitative demonstration of anabolic utilization of glucose and proline for biomass generation in remodeled pulmonary vessels, including colocalization with collagen fibrils — one putative endpoint of pathologic metabolic reprogramming. Given the central importance of glucose and aa to cellular homeostasis and the highly interconnected nature of such pathways, it remains unknown whether metabolic dependencies in PAH can be therapeutically targeted with sufficient efficacy and specificity. Nonetheless, this study demonstrates a template of how to apply MIMS to study the interplay between metabolism and disease pathology, which can also be applied to test the efficacy of potentially novel genetic or pharmacological manipulations in model organisms and human patients with PAH.

## Methods

Please refer to the online Supplemental Methods for additional details on study methods.

### Constructing the networks.

The consolidated human protein–protein interactome was assembled from different resources as described before, containing 16,470 proteins and 233,957 interactions (19, 23, 24). We mapped the differentially expressed rat genes to human orthologs using the HGNC Comparison of Ortholog Predictions search tool (https://www.genenames.org/tools/hcop/) and obtained 2,626 human genes. We then mapped these human genes to the consolidated human interactome and constructed a network of 1,836 proteins, and 6,748 interactions. Genes associated with aa were from the GO database (http://geneontology.org/). Genes associated with PAH were compiled from different resources (19, 23, 24) and mapped to the human interactome to form a PAH disease module. Genes associated with pathophenotypes were compiled from Phenopedia and DisGeNET (52, 53). The bipartite networks between glucose/proline and PAH were generated by retaining interactions between glucose/proline genes and PAH genes in the human interactome.

### Calculating network proximity.

We used network proximity to quantify the closeness of aa pathways to the PAH module (19, 23, 24). Network proximity is defined as the average minimum shortest path length in the interactome from an aa gene to the PAH disease module:
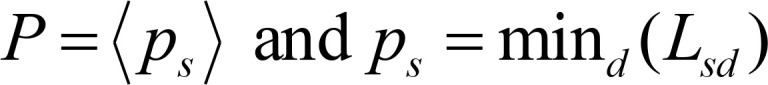


where *p*_s_ is the minimum shortest path length in the human interactome from an aa gene *s* to the PAH disease module.

### MIMS.

Stable isotope tracers (Cambridge Isotope Laboratory) consisting of ^2^H-glucose (250 mg per dose) and ^15^N-proline (25 mg per dose) were administered to monocrotaline- (MCT-) or vehicle-treated rats by i.p. injection twice in the 24 hours prior to sacrifice. The lungs were perfused with 4% paraformaldehyde, embedded in EPON resin, sectioned to 0.5 microns, and mounted on silicon wafers. Samples were then analyzed with a NanoSIMS 50L instrument (CAMECA), using previously published analytical methods (10, 12, 35). ^15^N-proline was quantified by measuring the ^12^C^15^N^–^/^12^C^14^N^–^ ratio as described previously (10, 17) and ^2^H-glucose was quantified by measuring the ^12^C_2_^2^H^–^/^12^C_2_^1^H^–^ ratio as described previously (12, 35). The instrument was also tuned to capture ^31^P^–^ and ^32^S^–^. Image files were visualized and analyzed with a custom plug-in to ImageJ (NIH): OpenMIMS 3.0: https://github.com/BWHCNI/OpenMIMS (commit ID af175e) (12). Isotope ratio data were shown as HSI images. The lower bound of the scale (blue) was set at natural background as verified by analysis of unlabeled samples and/or embedding resin (e.g., for ^15^N-data, a lower bound of 0 is equivalent to the natural background of 0.37% and an upper bound of 100 would correspond to a ratio of 0.74% or 2-fold enrichment). The upper bound of the scale was set to demonstrate regional differences in enrichment. While scaling changes affect the color pattern, the underlying quantitative data remain unmodified.

### Statistics.

All statistical analyses were performed using Origin 9.01 or OriginPro, GraphPad Prism v7.03 or v9.4, Cytoscape 3.5.1, and R 4.0.3 with the ggpubr 0.4.0 and tidyverse 1.3.0 packages. The significance of network proximity was evaluated by creating 1,000 random modules of the same size and comparing the observed proximity value with the null model (random control) through fitting normal distributions and *P* values were obtained by *z* test. Data are presented as the mean ± SEM unless otherwise indicated. Comparison between 2 groups was performed using the Student’s unpaired 2-tailed *t* test. The paired 2-tailed Student’s *t* test was used for analyses comparing metabolic tracer signal differences in the pulmonary endothelium and vascular media within the same rat. Hypergeometric testing was applied using the fgsea R package to identify key MSigDB Hallmark pathways distinguishing control versus inflammatory PAH (as described further in the legend of Supplemental Figure 3B). The Mann-Whitney and Kruskal-Wallis nonparametric tests were used to compare 2 or more non-normally distributed groups. Cell type–specific differences in ^15^N-proline or ^2^H-glucose labeling between MCT and control rats were assessed by nested ANOVA. The Pearson or Spearman (for experiments with small sample size) correlation coefficient is reported for linear regression. Graphical representation of comparisons including *N* ≥ 3 uses the box or violin plot format inclusive of mean, median, IQR, and maximum and minimum. A *P* value < 0.05 and FDR < 0.05 were considered statistically significant.

All data were included for analysis with the following exception: the right ventricular systolic pressure could not be measured in 1 rat due to periprocedural mortality from hemorrhage. MIMS and accompanying histologic experiments used 3 biological replicates per condition. No nonlinear adjustments were made to representative images. Primary data were reviewed in a blinded manner when possible.

### Study approval.

All experiments involving animals followed established protocols that were approved by the IACUC at Brigham and Women’s Hospital (Protocol 2016N000401). Procedures involving animal welfare were performed in line with guidelines established by the Panel on Euthanasia of the American Veterinary Medical Association.

## Author contributions

BMW designed the research studies, conducted experiments, acquired data, analyzed data, provided reagents, and edited the manuscript. RSW acquired data, analyzed data, and edited the manuscript. CG conducted experiments, acquired data, and analyzed data. CVRH analyzed data, assembled video files, and edited the manuscript. WMO reviewed data and edited the manuscript. JM analyzed data, assembled video files, and edited the manuscript. MLS designed research studies, conducted experiments, acquired data, analyzed data, provided reagents, and assembled and edited the manuscript. BAM designed research studies, conducted experiments, acquired data, analyzed data, provided reagents, and assembled and edited the manuscript. Senior authors MLS and BAM reviewed primary data using blinding when possible. All authors had access to the primary data.

## Supplementary Material

Supplemental data

Supplemental video 1

Supplemental video 2

## Figures and Tables

**Table 1:**
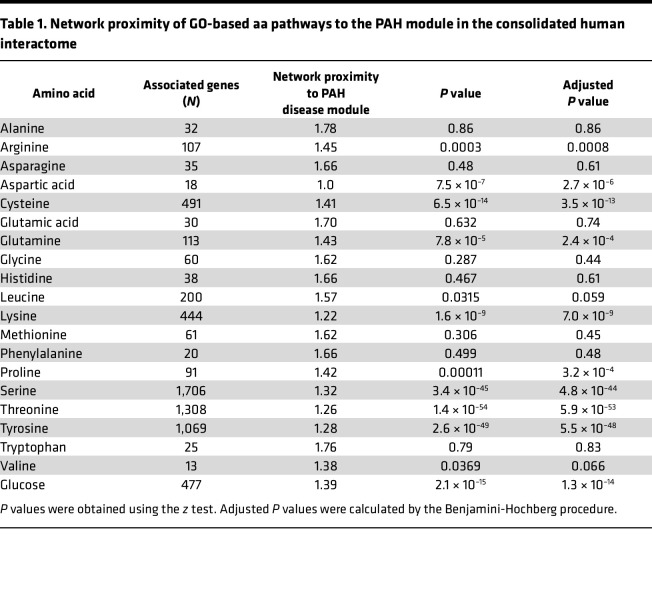
Network proximity of GO-based aa pathways to the PAH module in the consolidated human interactome

**Table 2:**
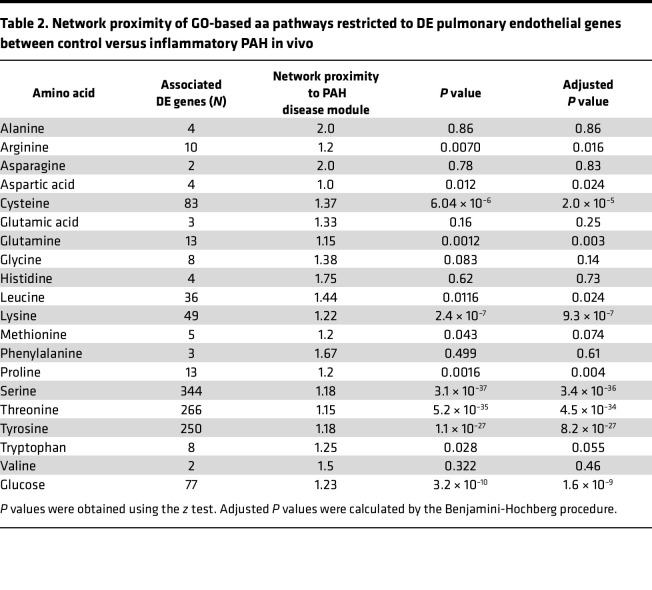
Network proximity of GO-based aa pathways restricted to DE pulmonary endothelial genes between control versus inflammatory PAH in vivo

**Table 3:**
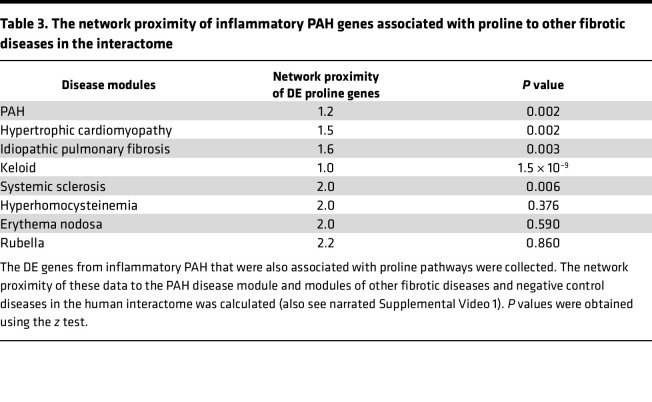
The network proximity of inflammatory PAH genes associated with proline to other fibrotic diseases in the interactome

## Version 1 01/10/2023

In-Press Preview

## Version 2 02/22/2023

Electronic publication
