# Supplementary material for: Proline and glucose metabolic reprogramming supports vascular endothelial and medial biomass in pulmonary arterial hypertension
Source: JCI Insight. 2023 Feb 22;8(4):e163932. doi: 10.1172/jci.insight.163932 (PMC9977503; doi:10.1172/jci.insight.163932)
Supplement: Supplemental data [file jciinsight-8-163932-s074.pdf]

Proline and glucose metabolic reprogramming supports

vascular endothelial and medial biomass in pulmonary arterial hypertension

Supplementary Methods

Inflammatory model of PAH and administration of tracer in vivo. Male Sprague-Dawley rats (12-14 weeks of age; Charles River Laboratory) were administered a single intraperitoneal injection of monocrotaline (MCT) (50 mg/kg)(Sigma) and tissue harvesting was performed 23-25 d later, as reported previously (1-3). Rats were injected with stable isotope tracer consisting of 250 mg ^{2}H-glucose and 25 mg ^{15}N-proline approximately 24 and 12 hours prior to sacrifice via the intraperitoneal route. Animals were allowed access to water and standard chow ad libitum.

Rat lung histology. In pilot experiments, hematoxylin/eosin and picrosirius red stain was performed to confirm the inflammatory arteriopathy that is pathognomonic for MCT-PAH. Briefly, lung samples were perfused with 10% neutral-buffered formalin at a pressure of 20 cm H_2O prior to harvesting(1-3) and then fixed with formalin for at least 24 hr at room temperature. Samples were processed/embedded in paraffin using a Hypercenter XP System and Embedding Center (Shandon). The paraffin-embedded lung tissue was cut into 5-µm sections and stained with picrosirius red (Polysciences), according to manufacturer's instructions. Qualitative assessment of vascular remodeling and, thus, confirmation that the model was initiated successfully was performed by two PAH experts (B.M.W., B.A.M.) prior to proceeding with MIMS analyses. To quantify arteriolar fibrosis, brightfield microscopy images were captured at 630X total magnification on a (Zeiss AxioImager A2), and vascular wall collagen was determined using the ImageJ software plugin MRI_Fibrosis_Tool (https://github.com/MontpellierRessourcesImagerie/imagej_macros_and_scripts/wiki/MRI_Fibrosis_Tool). Vascular collagen was expressed as Percent Luminal Fibrosis: ([total area of collagen signal in the vessel

wall]/[Area of the lumen]) x 100. In corollary experiments using the same MCT lot and procedural methods detailed above, we also performed cardiac catheterization to confirm pulmonary hypertension was evident in the MCT-PAH models, as reported extensively by our group (1-3) and others (4) previously (**Figure S11 and S13**).

Rat lung immunofluoresence. Rat lung specimens were harvested and processed as described above. Formalin-fixed paraffin embedded tissue sections (5-µm) were deparaffinized and rehydrated (xylene for 5 min x 2, 100% ethanol for 1 min x 2, 95% ethanol for 1 min x 2) and subjected to high temperature antigen retrieval in a vegetable steamer for 20 min using a citrate-based antigen retrieval solution (#BM-745, Boston BioProducts). Sections were blocked in 10% goat serum with 1% BSA in PBS for one hour at room temperature and then incubated with primary antibody (**Table S3**) at the specified dilution in 1% BSA in PBS at 4° C overnight. When applicable, species- and concentration-matched IgG control was used to test for nonspecific labeling. Sections were washed in PBS for 15 min and then incubated with secondary antibody **(Table S3)** at the specified dilution for one hour at room temperature. Slides were then washed in PBS for 1 min, incubated with DAPI (300 nM in PBS, #D1306, Invitrogen) for 25 min, washed for 10 min in PBS, air dried, and mounted (ProLong DiamondAntifade, #P6970, Invitrogen). Slides were imaged at 100X and 400X total magnification on a Zeiss LSM800 confocal microscope using the Airscan Frame mode.

Pulmonary artery endothelial cell (PAEC) and RNA isolation ex vivo. The detailed methods for isolating PAECs from rat lungs have been published previously by our laboratory (5). Briefly, vehicle or MCT-treated rats were sacrificed, and the lungs were flushed with PBS until clear of blood. Peripheral lung tissue was excised, mechanically dissociated, enzymatically digested, strained, and centrifuged to produce a cell pellet. Next, the cell pellet was subjected to immunomagnetic bead selection for CD31-positive cells. The recovered CD31-positive cells were released from magnetic beads, labeled with anti-CD31 antibody (#FAB3628P, R&D Biosystems, 20 µg/mL) and Griffonia simplicifolia isolectin-B4 (GS-IB4) (#I214**11,**

Thermo Fisher, 5 μg/mL) and prepared for fluorescence-activated cell sorting (FACS). Using FACS (FACS Aria Special Order flow cytometer), intact cells (as assessed by propidium iodide) demonstrating high CD31 and GS-IB_4 signal were sorted into cold Trizol LS (Thermo Fisher, #10296010) for immediate RNA isolation. RNA was then assessed for quality using an Agilent 2100 Bioanalyzer and found to have RNA integrity number 9.2 ± 0.1 vs. 8.7 ± 0.1, control vs MCT, respectively, as reported in a previous publication (5).

RNA-Seq and transcriptomic analyses. Our pipeline for RNA sequencing and analysis has been reported previously (1). In summary, sequencing libraries were generated by poly-A selection of mRNA followed by cDNA generation using the Illumina TruSeq kit. Library quality was confirmed by Agilent DNA High Sensitivity Chip and RT-PCR. Sequencing was performed on an Illumina HiSeq 2500 to a depth of approximately 40-50 million paired-end reads. The FASTQC (https://github.com/s-andrews/FastQC) and FASTX (https://github.com/agordon/fastx_toolkit) software packages were used to clean the sequencing data. Alignment to the human genome was performed using the tophat2 and bowtie2 packages (6).

In this dataset, there are 25,808 genomic features across N=12 pulmonary artery endothelial cell samples (N=6 control samples and N=6 PAH samples). After removing those genomic features that did not have any counts in any samples, there were N=19,392 genomic features remaining for further analysis. Normalized count data were generated using the EdgeR software package to identify differentially expressed genes in PAH vs control as defined by false discovery rate (FDR) <0.05 (8) (accession ID, GSE221097).

Gene set enrichment analysis: Human orthologs of differentially expressed genes between control and PAH PAECs were identified as above. Gene set enrichment was performed on differentially expressed genes using MSigDB Hallmark pathway list (9,10) using statistical methods described in the *Statistical Methods* section of the main manuscript. A pathway was considered enriched if it satisfied FDR<0.05.

Overlap with other inflammatory conditions: Genes associated with common inflammatory diseases were assembled from DisGeNET and then mapped to the human interactome. Next, hypergeometric testing was applied to evaluate the significance of the number of genes shared between each inflammatory disease and differentially expressed human orthologs between control and PAH PAECs using the number of disease genes present in the interactome as background. Significant overlap was observed if $P<0.05$.

Assembling video figures of the interactome and disease modules: A low dimensional embedding of the human interactome composed of 16,470 proteins and 233,957 protein interactions was assembled using VRNetzer, a Virtual Reality (VR) Network analytics and visualization platform (11). Due to the high-dimensional, complex nature of networks, a meaningful representation in low dimensions is dependent on research focus and may be used to communicate findings as well as to visually explore unknown territory to form hypotheses to follow up on statistically. The network layout chosen in the current work captures the global architecture of the complex network (12) and is modulated to additionally express the statistical observations within the lower dimensional embedding to be reflected in node proximities. Through incorporation of statistical observations into a matrix of visiting probabilities, as the foundation of the three-dimensional network representation, respective statistically observed distances of disease modules and pathway protein groups can be communicated locally and placed within the context of the large network.

In the video files, the network proximity of glucose and proline pathways to the PAH disease module is shown when positioned into three-dimensional space, which is based on the incorporation of statistical values into the embedding process. This approach allows for locally communicated findings on spatial relationships between pathways, as well as to visually explore the modulated subsets of proteins (i.e., nodes) within the global context of the whole consolidated human interactome. The Bipartite connected subgraph emphasizes the identified overlap of pathway proteins and the disease modules visually.

Supplementary References

1. Samokhin AO, Stephens T, Wertheim BM, Wang RS, Vargas SO, Yung LM, et al. NEDD9 targets COL3A1 to promote endothelial fibrosis and pulmonary arterial hypertension. *Sci Transl Med*. 2018;10(445).
2. Maron BA, Zhang YY, White K, Chan SY, Handy DE, Mahoney CE, Loscalzo J, Leopold JA. Aldosterone inactivates the endothelin-B receptor via a cysteinyl thiol redox switch to decrease pulmonary endothelial nitric oxide levels and modulate pulmonary arterial hypertension. *Circulation*. 2012;126(8):963-74.
3. Aghamohammadzadeh R, Zhang YY, Stephens TE, Arons E, Zaman P, Polach KJ, Matar M, Yung LM, Yu PB, Bowman FP, Opotowsky AR, Waxman AB, Loscalzo J, Leopold JA, Maron BA. Up-regulation of the mammalian target of rapamycin complex 1 subunit Raptor by aldosterone induces abnormal pulmonary artery smooth muscle cell survival patterns to promote pulmonary arterial hypertension. *FASEB J*. 2016;30(7):2511-27.
4. Yung LM, Nikolic I, Paskin-Flerlage SD, Pearsall RS, Kumar R, Yu PB. A Selective transforming growth factor-β ligand trap attenuates pulmonary hypertension. *Am J Respir Crit Care Med*. 2016;194(9):1140-1151.
5. Wertheim BM, Lin Y-D, Zhang Y-Y, Samokhin AO, Alba GA, Arons E, Yu PB, Maron BA. Isolating pulmonary microvascular endothelial cells ex vivo: Implications for pulmonary arterial hypertension, and a caution on the use of commercial biomaterials. *PLoS One*. 2019;14(2):e0211909.
6. Kim D, Pertea G, Trapnell C, Pimentel H, Kelley R, Salzberg SL. TopHat2: accurate alignment of transcriptomes in the presence of insertions, deletions, and gene fusions. Genome Biol 2013;14(4):R36.
7. Langmead B, Salzberg SL. Fast gapped-read alignment with Bowtie 2. *Nat Methods*. 2012;9(4):357-9.
8. Robinson MD, McCarthy DJ, Smyth GK. edgeR: a Bioconductor package for differential expression analysis of digital gene expression data. *Bioinformatics*. 2010;26:139-40.
9. Korotkevich G, Sukhov V, BUdin N, Shpak B, Artyomov MN, Sergushichev A. Fast gene set enrichment analysis. BioRxiv 2021; 060012. https://doi.org/10.1101/060012

10. Liberzon A, Birger C, Thorvaldsdottir H, Ghandi M, Mesirov JP, Tamayo P. The molecular signature database (MSigDB) hallmark gene set collection. *Cell Syst*. 2015;1(6):417-425.

11. Pirch S, Mueller F, Iofinova E, Pazmandi J, Huetter CVR, Chiettini M., et al. The VRNetzer platform enables interactive network analysis in Virtual Reality. *Nat Commun*. 2021(12):2432.

12. Huetter, CVR, Sin C, Mueller F, Menche J. Network cartographs for interpretable visualizations. Nat Comput Sci 2022(2):84–89.

13. Guillermier C, Doherty SP, Whitney AG, Babaev VR, Linton MF, Steinhauser ML, et al. Imaging mass spectrometry reveals heterogeneity of proliferation and metabolism in atherosclerosis. *JCI Insight*. 2019;4(11): e128528.

Supplementary Figure Legends

Supplemental Figure 1. Gating strategy for the isolation of rat pulmonary artery endothelial cells by flow cytometry.

Supplemental Figure 2. The pulmonary artery endothelial expression profile in control and inflammatory PAH rat lungs in situ.

Supplemental Figure 3. Protein-protein interaction network representing genes that were differentially expressed in inflammatory PAH in vivo and observed in the human PAH module of the consolidated interactome.

Supplemental Figure 4. Network proximity to PAH module by individual amino acid and glucose pathway.

Supplemental Figure 5. Proline and glucose pathways in pulmonary arterial hypertension (PAH).

Supplemental Figure 6. Imaging of ^{15}N-proline utilization in rat lung with MIMS.

Supplemental Figure 7. Development of a MIMS imaging pipeline for the pulmonary vasculature.

Supplemental Figure 8. Heterogeneous ^{15}N-proline labeling in the remodeled pulmonary arteriole.

Supplemental Figure 9. MIMS imaging of red blood cells (RBC) demonstrates uniform label delivery in control and inflammatory PAH rats.

Supplemental Figure 10. Inflammatory PAH in vivo is associated with fibrotic remodeling of pulmonary arterioles.

Supplemental Figure 11. Single cell resolution label quantification reveals heterogeneity of metabolism.

Supplemental Figure 12. Endothelial and medial glucose avidity in PAH.

Supplemental Figure 13. Proline and glucose transporter expression in pulmonary artery endothelial cells are associated with pulmonary hypertension in vivo.

Supplemental Video Figure 1. Three-dimensional visualization of the human interactome and the relationship between proline pathways and different disease modules, including pulmonary arterial hypertension (PAH). Narration performed by C.H.

Supplemental Video Figure 2. Three-dimensional visualization of the human interactome illustrating proline and glucose pathways and the proximity of proline + glucose pathways to the pulmonary arterial hypertension (PAH) disease module. Narration performed by C.H.

Supplemental Table 1. Antibodies used in immunofluorescence experiments.

Antibody	Vendor	Catalog number	Dilution
von Willebrand Factor	Abbexa	102082	1:100
Alpha smooth muscle actin	Abcam	5694	1:1000
Alpha smooth muscle actin	Sigma	A2547	1:1000
CD31	Abcam	182981	1:1000
Mouse IgG	Santa Cruz	3890	0.001-0.01 mg/mL
Rabbit IgG	Abcam	171870	0.0005-0.002 mg/mL
Goat anti-mouse Alexa Fluor 488	Abcam	1500113	1:200
Goat anti-rabbit Alexa Fluor 568	Abcam	175471	1:200

Antibody dilutions were prepared in 1% BSA in PBS. IgG control dilutions were prepared to match concentration the respective primary endothelial marker antibody.

Supplemental Table 2. Overlap between differentially expressed pulmonary endothelial genes in inflammatory PAH model in vivo with other inflammatory (positive control) diseases in the human interactome.

Inflammatory Disease Comparator	Genes (N) in Disease Module within the Interactome	MCT-PAH PAEC Human Orthologs	P-value
Systemic sclerosis	1204	289	P< 2.3e-21
Crohn’s disease	863	230	P< 3.9e-23
Myocarditis	255	64	P< 2.8e-06
Glomerulonephritis	364	110	P< 2.0e-15

P-values were determined by the hypergeometric test. MCT, monocrotaline; PAH, pulmonary arterial hypertension; PAEC, pulmonary artery endothelial cells.

Supplemental Table 3. The network proximity between proline genes collected from the Gene Ontology (GO) database and different disease modules within the consolidated human interactome.

Disease modules	Network Proximity of Proline Genes from GO	P-value
PAH	1.42	0.0001
HCM	1.69	3.2 e-05
Idiopathic pulmonary fibrosis	1.87	0.0236
Keloid	1.47	5.1 e-09
Systemic Sclerosis	2.14	0.002

P-values were calculated by the z-test. HCM, hypertrophic cardiomyopathy.

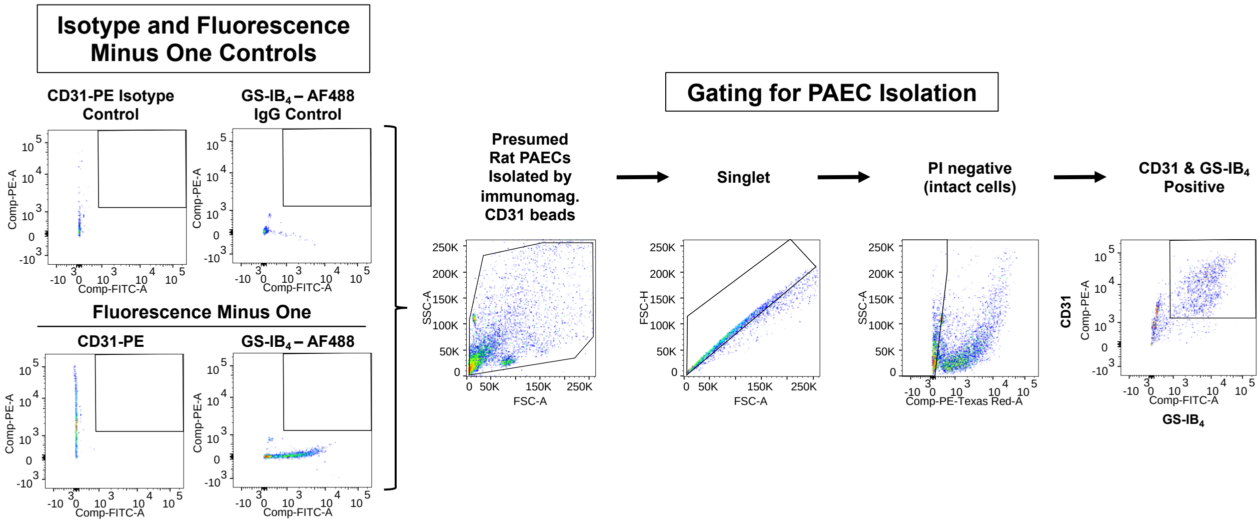

Supplemental Figure 1. Gating strategy for the isolation of rat pulmonary artery endothelial cells by flow cytometry. Rats were treated with 50 mg/kg monocrotaline or vehicle control by intraperitoneal injection. On day 23, peripheral lung tissue was excised and prepared into a single cell suspension. CD31-positive cells were labeled with a different CD31 antibody and *Griffonia simplicifolia* isolectin B_4 and subjected to fluorescence activated cell sorting using gates established through the use of isotype and fluorescence minus one controls. Only intact (PI negative) cells positive for CD31 and GS-IB_4 were selected for transcriptomic analysis. Abbreviations: AF488, Alexa Fluor 488; GS-IB_4, FSC-A, forward scatter-area; *Griffonia simplicifolia* isolectin $B_{4;}$ PAEC, pulmonary artery endothelial cell; PE, phycoerythrin, PI, propidium iodide; SSC-A, side scatter, area.

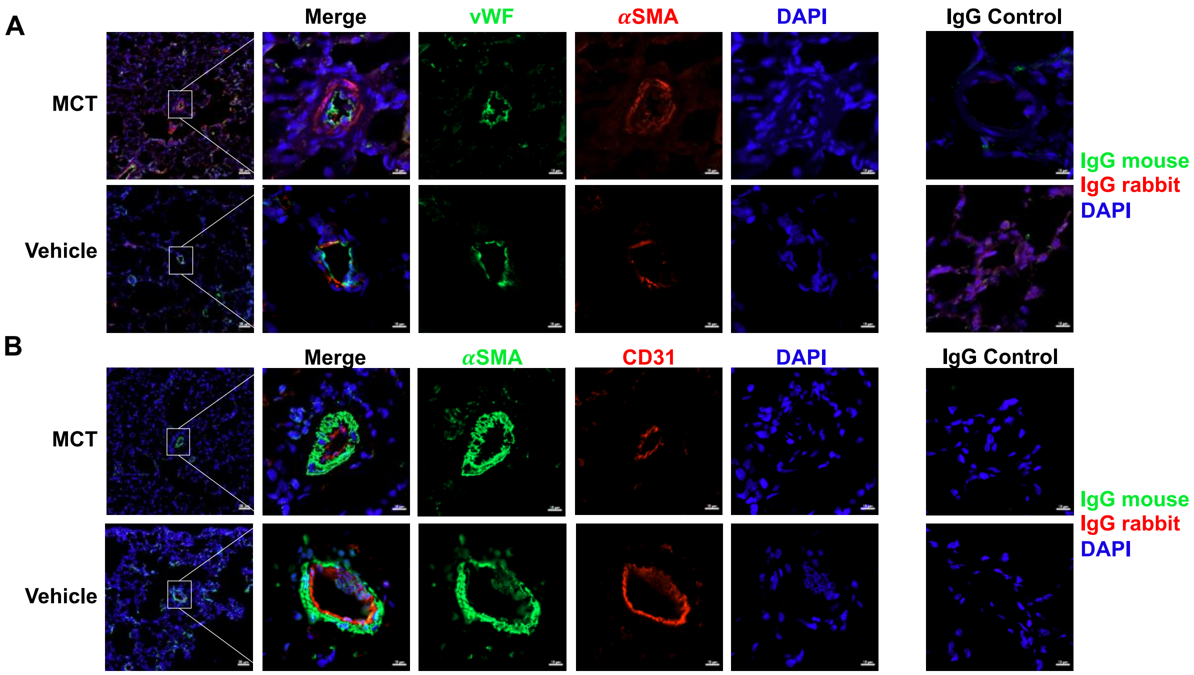

Supplemental Figure 2. Immunofluorescence against endothelial markers identifies a cell monolayer anatomically located at the arteriolar luminal interface. Rats received 50 mg/kg monocrotaline by intraperitoneal injection or vehicle control and were sacrificed 23-25 days later. Formalin-fixed paraffin-embedded 5-micron sections were analyzed at 100X (scale bar = 50μm) and 400X (inset) (scale bar = 10μm) on a Zeiss LSM800 confocal microscope in Airyscan Frame mode. A) IF microscopy of von Willebrand factor (Abbexa #102082, 1:100 dilution) and α-smooth muscle actin (Abcam #5694, 1:1000 dilution) vs. IgG control (Mouse IgG, Santa Cruz #3890, 0.01 mg/mL and rabbit IgG Abcam #171870, 0.002 mg/mL). B) IF microscopy of CD31 (Abcam #182981, 1:1000 dilution) and α-smooth muscle actin (Sigma #A2547, 1:1000) vs IgG control (Mouse IgG, Santa Cruz #3890, 0.01 mg/mL and rabbit IgG Abcam #171870, 0.002 mg/mL Abbreviations: α-SMA, α-smooth muscle actin; IF, immunofluorescence; MCT, monocrotaline; vWF, von Willebrand Factor.

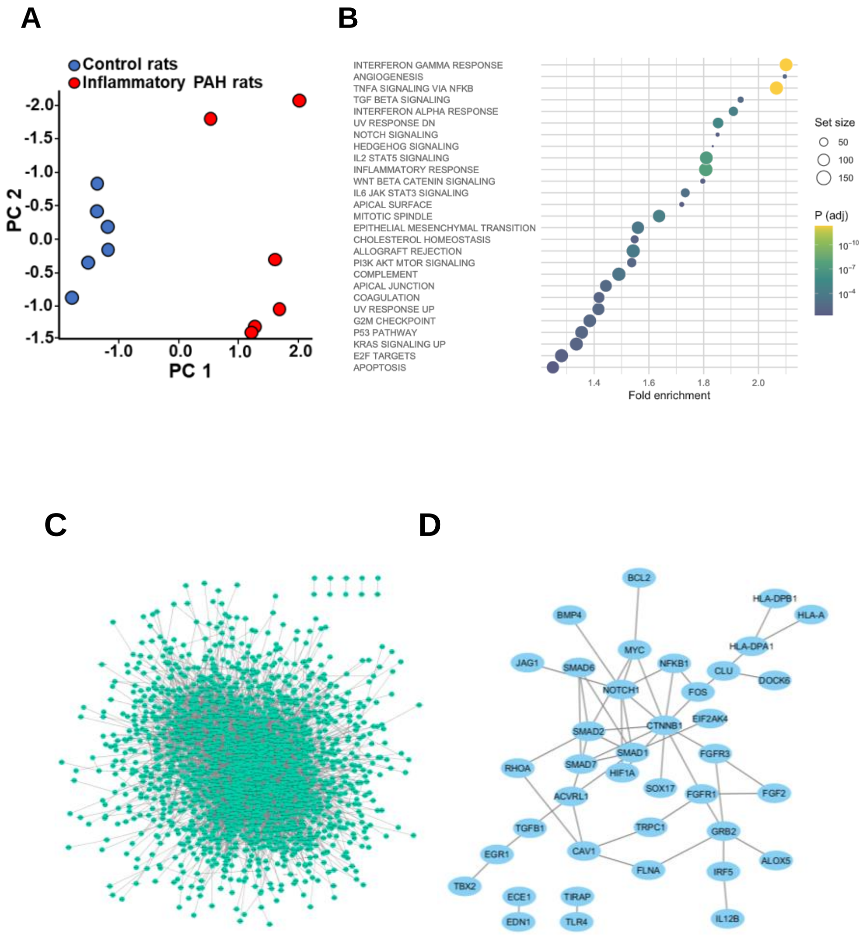

Supplemental Figure 3. Protein-protein interaction network representing genes that were differentially expressed in inflammatory PAH in vivo and observed in the human PAH module of the consolidated interactome. Pulmonary artery endothelial cells (PAECs) were isolated from control Sprague Dawley (SD) rats or SD rats administered monocrotaline (50 mg/kg) to induce inflammatory PAH in vivo (N=6 rats/condition). Differentially expressed genes from the PAECs are analyzed by RNA-Seq. **(A)** The transcriptomic profile by individual rat stratified by the first and second principal component vector. From a total of N=25,808 unique pulmonary endothelial genomic features captured by RNA-Seq analysis, we identified N=3,857 (14.9%) that were differentially expressed between control and inflammatory PAH rats (False discovery rate [FDR]<0.05; P<1.0 e-4). **(B)** Hypergeometric testing was applied to differentially expressed PAEC genes (FDR <0.05) to identify key MSigDB Hallmark pathways distinguishing control vs. inflammatory PAH. Enrichment analysis confirmed an inflammatory PAEC phenotype and reinforced model and analytic validity. We identified multiple pro-fibrotic Hallmark pathways including Epithelial to Mesenchymal Transition, TGF-β signaling, E2F targets, and other pathways regulating metabolism and fibrotic potential of PAECs in PAH. TGF, transforming growth factor; IL, interleukin; UV, ultraviolet; DN, down regulated. **(C)** To identify novel metabolic drivers of PAEC remodeling, differentially expressed genes were then mapped to the human interactome, resulting in a network of functionally important protein-protein interactions (PPIs) that included N=1836 proteins and N=6,748 interactions. **(D)** PPI network representing differentially expressed genes between control vs. inflammatory PAH also identified within the human PAH module of the interactome.

A

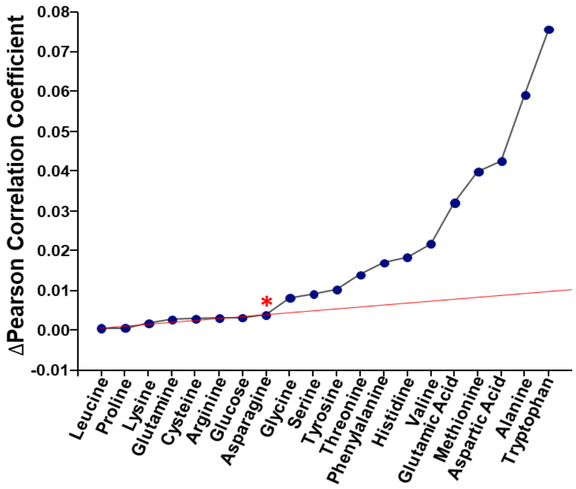

B

PPI in the proline-PAH bipartite network	
**Proline Gene (from PAH in vivo)**	**Glucose Gene Forming Edge**
YAP1	PDK1
ABL1	PDK1
PFN1	CMA1
FKBP7	ADIPOQ
ABL1	TERT
ABL1	MYC
YAP1	EGR1
CCND1	CDKN1B
ABL1	CDKN1B
PFN1	SRC
ABL1	SRC
ABL1	MTOR
PFN1	RHOA

Supplemental Figure 4. Network proximity to PAH module by individual amino acid and glucose pathway. The proximity to the pulmonary arterial hypertension (PAH) module for pathways regulated by each amino acid and glucose collected from (i) gene ontology and (ii) differentially expressed genes in the pulmonary endothelium of PAH rats compared to control rats in vivo was analyzed by univariate linear regression. **(A)** The effect on the Pearson correlation coefficient after removing each individual amino acid is plotted. Divergence from the linear regression is observed beginning with glycine (asterisk) and continued for entries through tryptophan. These data indicate strongest agreement in proximity to the human PAH module for pathways regulated by leucine, proline, lysine, glutamine, cysteine, arginine, glucose, and asparagine. **(B)** The proline nodes that were differentially expressed in PAH in vivo and connected to a glucose node in the PAH module in the interactome (see Figure 1E for details). PPI, protein-protein interaction.

A

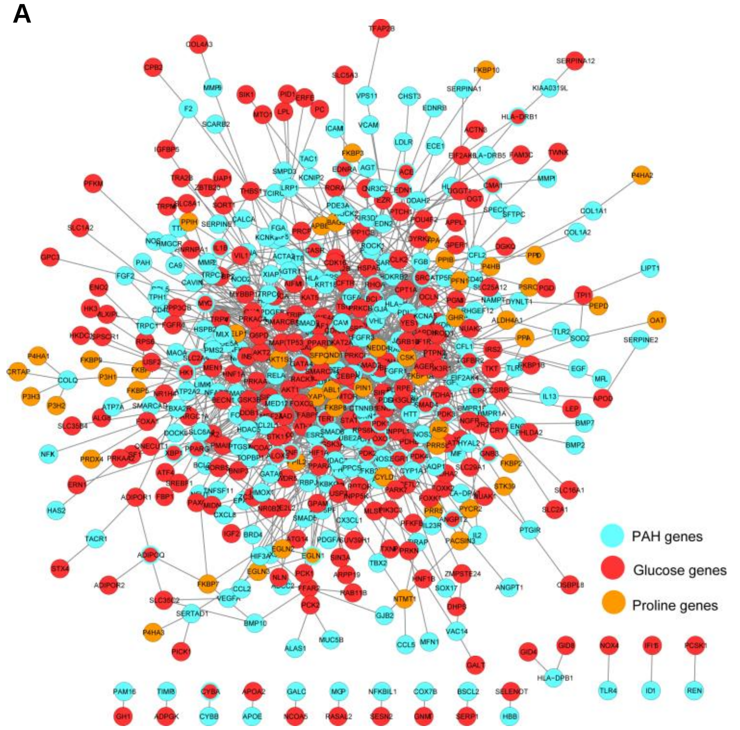

B

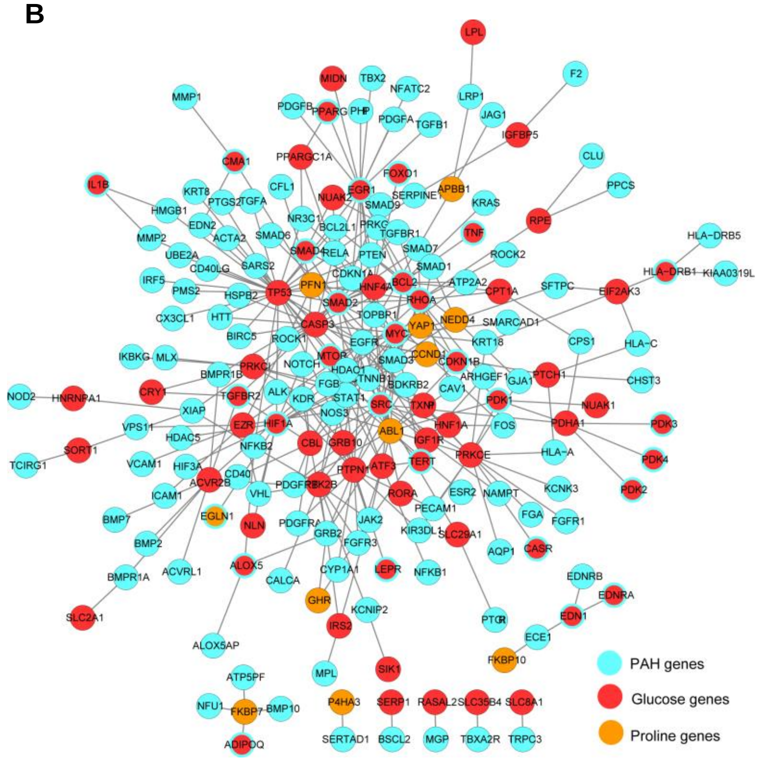

Supplemental Figure 5. Proline and glucose pathways in pulmonary arterial hypertension (PAH). (A) The PAH disease module stratified by gene ontology (GO) proline and glucose pathways. **(B)** The PAH disease module stratified by GO proline and glucose pathways, restricted to include only genes that were DE in PAH in vivo. These data an expanded view from Figure 1D.

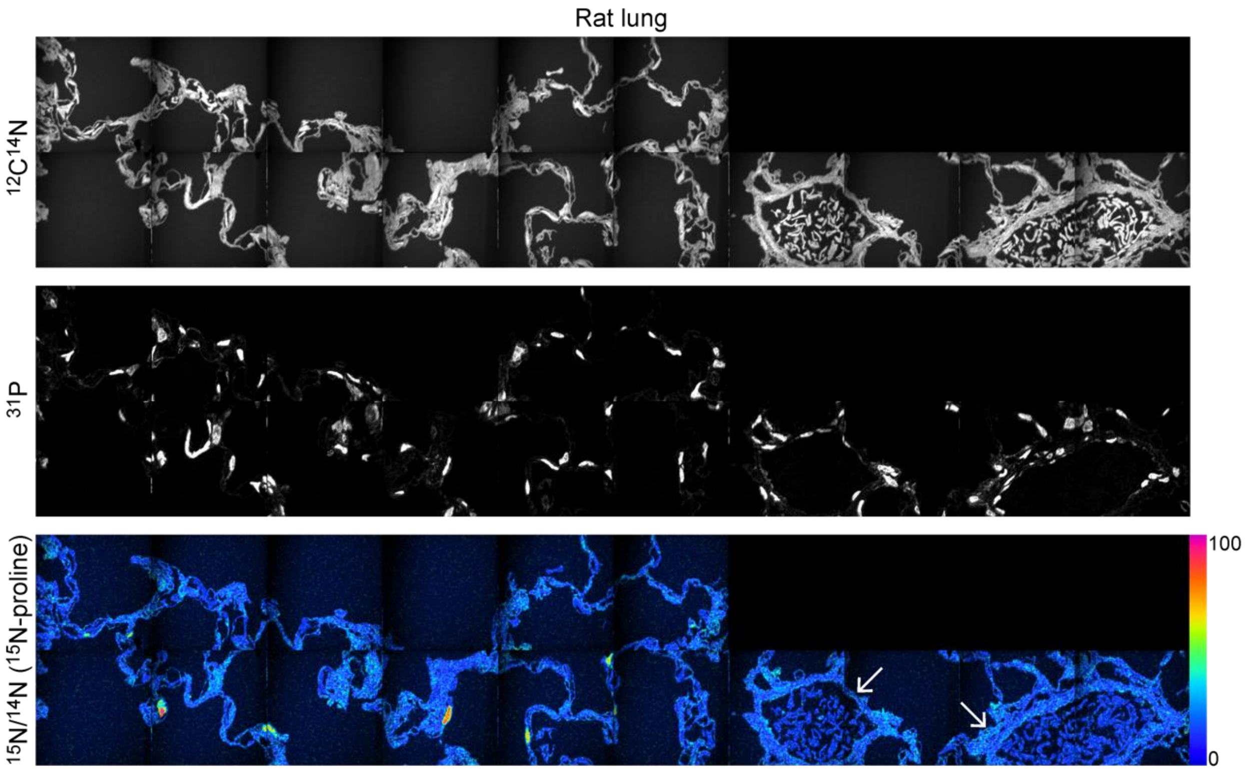

Supplemental Figure 6. Multi-isotope imaging mass spectrometry (MIMS) analysis was conducted in chain analysis mode and the imaging fields subsequently stitched together to make a “mosaic image.” Top: $^{12}C^{14}N$ mass image demonstrates stereotypical lung histological features and two blood vessels containing red blood cells. Middle: ^{31}P mass image demonstrates nuclei due to the high phosphorus content of chromatin. Bottom: $^{12}C^{15}N/^{12}C^{14}N$ hue saturation intensity (HSI) image demonstrates ^{15}N-proline labeling, including in the walls of the blood vessels (arrows) indicated by the turquoise coloring. The scale is set such that the bottom end of the scale is set to natural abundance (0.37% for ^{15}N – no labeling) and the upper bound of the scale is set at 0.74% (100% above background).

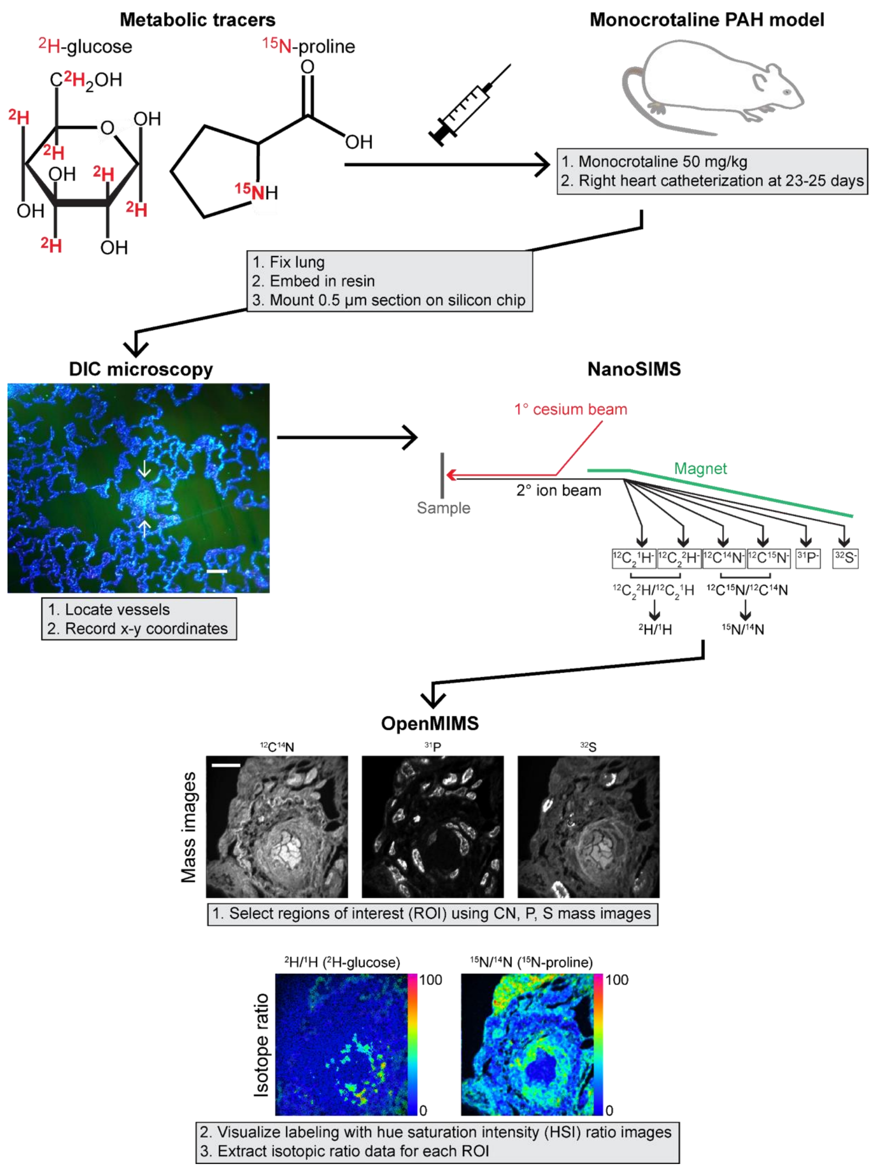

Supplemental Figure 7. Experimental workflow for multi-isotope imaging mass spectrometry (MIMS) analysis of pulmonary vessels. Metabolic tracers: Glucose enriched in the rare stable isotope of hydrogen (^{2}H) and proline enriched in the rare stable isotope of nitrogen (^{15}N) were used as tracers. Monocrotaline PAH model: Tracers were administered by intraperitoneal injection to a rat model of pulmonary arterial hypertension (PAH), in which the disease had been induced by administration of monocrotaline (MCT). Dose selection was based on prior experience with glucose and other amino acid tracers in mouse models, with weight-based dose conversion for the rat. At sacrifice, the lungs were fixed, embedded in plastic resin, sectioned, and mounted on silicon chips. DIC microscopy: The opaque silicon chip is analogous to the glass microscope slide used for light microscopy and can be imaged by differential interference contrast (DIC) microscopy. Vessels were located using the DIC scope. NanoSIMS: The NanoSIMS instrument was tuned to capture the ionic masses of interest. The top-most layer of the surface is destructively sputtered with a focused primary beam of cesium ions whose ultimate size (below 50 nm) allows for exploration of samples at sub-cellular resolution. A fraction of the constituent atoms and small molecular fragments are ionized to become secondary ions. Negatively charged secondary ions are extracted and guided to a magnetic sector mass spectrometer, where up to seven detectors are tuned to count ions of interest in parallel. Analysis of a spot on a sample surface forms the quantitative basis for a single pixel of a quantitative mass image. Consequently, a 256 x 256 pixel image is based on measurements of 65,536 spots (i.e., 256 x 256=65,536 spots) and a 512 x 512 pixel image is based on measurement of 262,144 spots. OpenMIMS: The quantitative imaging files were accessed in a custom plugin to ImageJ called OpenMIMS. Mass images used to reveal histological details (^{12}C^{14}N, ^{31}P, and ^{32}S) were used to identify cells and vessel structures of interest, which were manually traced to generate regions of interest (ROI). The identify of these structures/cells was assigned by an observer blind to the ratio data. The ratio images, which indicate labeling, were then constructed and visualized. The isotope ratios for each ROI were then extracted. Scale=10 um.

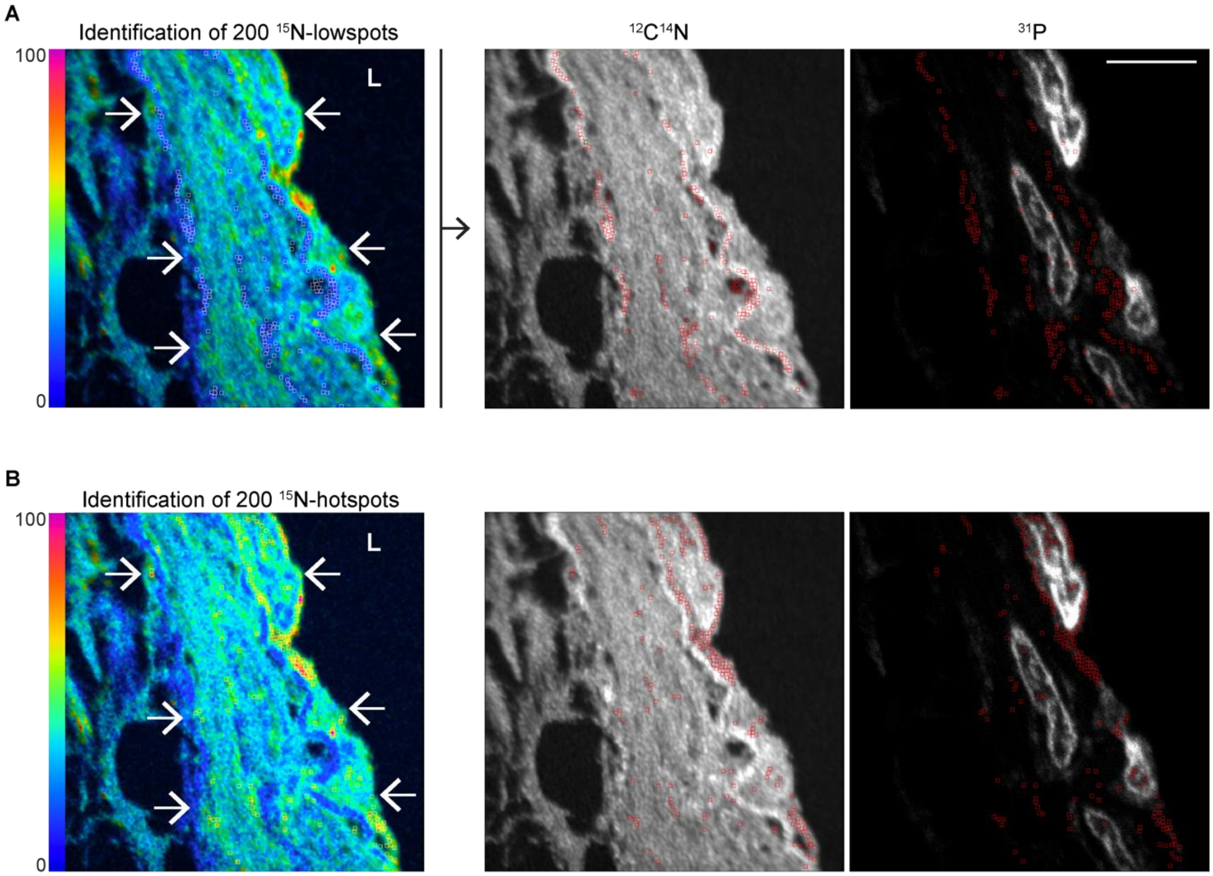

Supplemental Figure 8. Heterogeneous ^{15}N-proline labeling in the remodeled pulmonary arteriole. Analysis of the multi-isotope imaging mass spectrometry (MIMS) hue, saturation, intensity (HSI) ratio image shown in Figure 2. (A) In the area of the vessel wall contained by the arrows, the 200 spots (5 pixels X 5 pixels) with the lowest ^{15}N/^{14}N ratio were selected in unbiased fashion using OpenMIMS. These spots are contained in the white boxes in the HSI image (left) and in red boxes in the other mass images. The "low-spots" are concentrated along the elastin bands as visualized in the ^{12}C^{14}N image (middle). There are scant low-spots in the nuclei and concentrated in the ^{31}P-rich chromatin (right). (B) In the area of the vessel wall contained by the arrows, the 200 spots (5 pixels X 5 pixels) with the highest ^{15}N/^{14}N ratio were selected in unbiased fashion using OpenMIMS and displayed as in A. The hotspots are concentrated in the extra-nuclear regions of the endothelium and scattered throughout the vessel wall. Lumen, L. Scale=5 um.

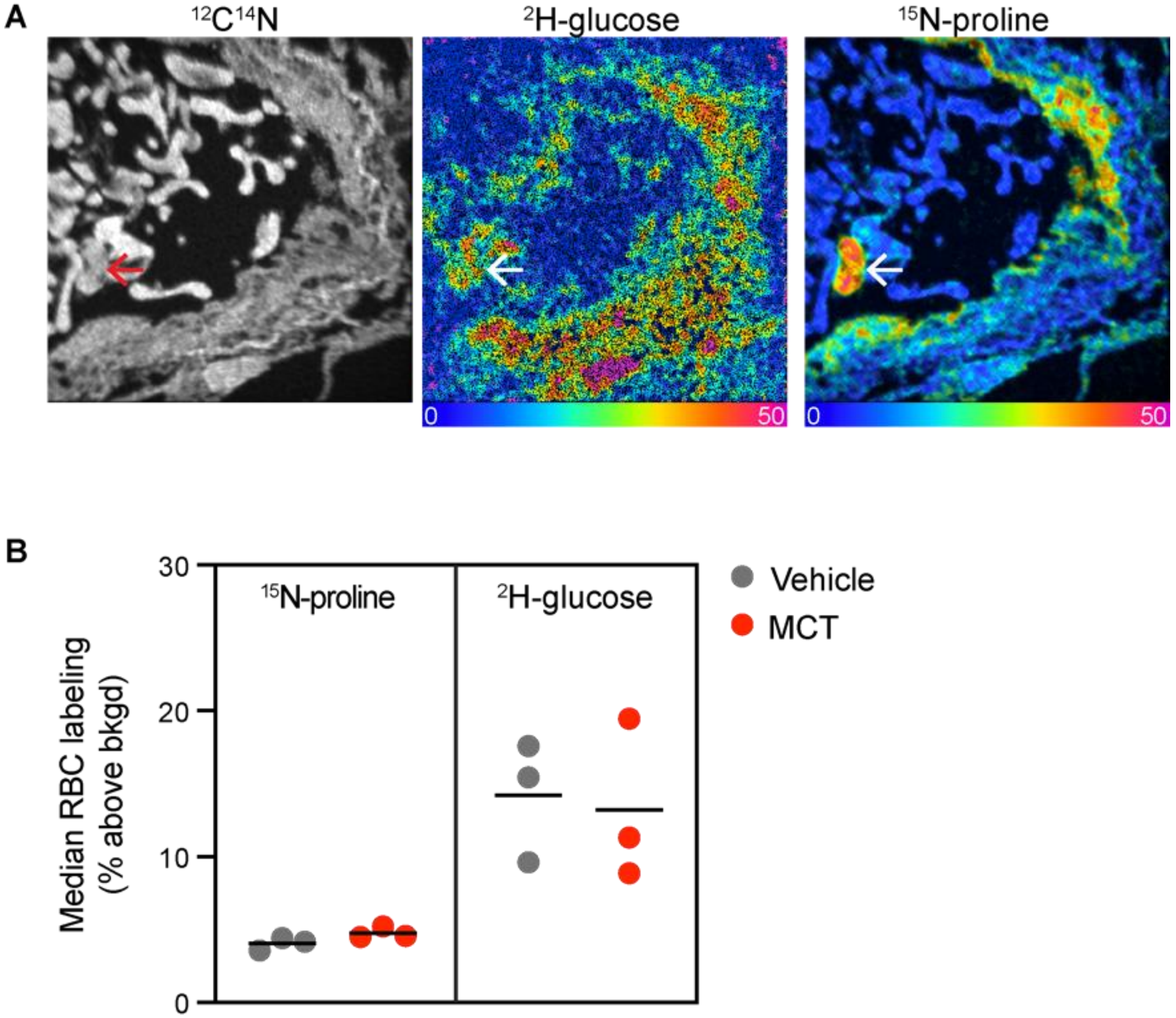

Supplemental Figure 9. Multi-isotope imaging mass spectrometry (MIMS) imaging of red blood cells (RBC) demonstrates uniform label delivery in control and inflammatory pulmonary arterial hypertension (PAH) rats. (A) Pulmonary vessel from monocrotaline (MCT)-PAH rat. Stereotypical red blood cell (RBC) evident in the lumen of the vessel and have a non-normal labeling distribution due to scattered outliers, one of which is indicated by the arrow. It is labeled with ^{15}N-proline (right) and ^{2}H-glucose (middle). (B) The median RBC labeling for each rat is graphed for ^{15}N-proline (left) and ^{2}H-glucose (right). The degree of labeling is not significantly different between vehicle control and PAH RBC (unpaired Student t-test).

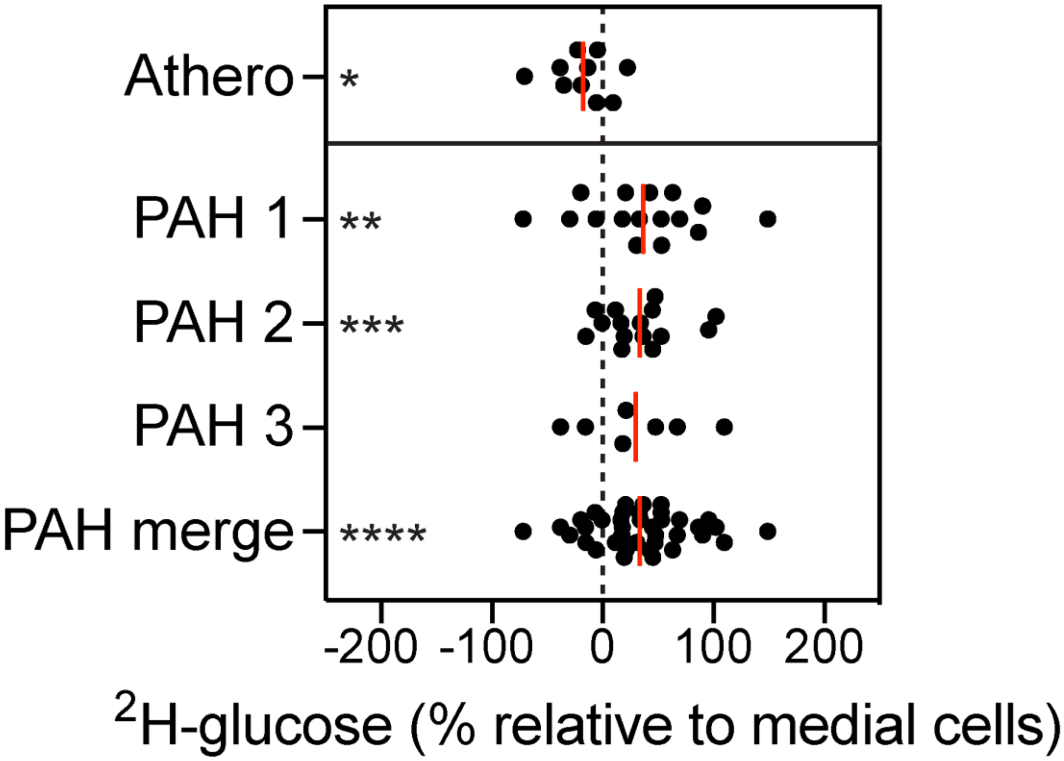

Supplemental Figure 10. Endothelial and medial glucose avidity in PAH. Dot plot of endothelial ^{2}H-glucose labeling normalized to the corresponding medial cells. In a prior study ^{2}H-glucose (but not ^{15}N-proline) was administered to a murine inflammatory atherosclerosis model (14). The endothelium was not a primary focus of that study, accounting for the modest number of analyzed endothelial cells; however, in a merged analysis there was a significant downward shift in endothelial ^{2}H-labeling normalized to medial cells from the same lesions. In contrast, PAH endothelial cells demonstrated augmentation of ^{2}H-labeling relative to medial cells, similar to what was observed with ^{15}N-proline (Figure 3F). Each dot indicates normalized ^{2}H-labeling relative to medial cells from the same vessel and the red line indicates the mean; *$p<0.05$; **$p<0.01$; ***$p<0.005$; ****$p<0.0001$, two-sided t test.

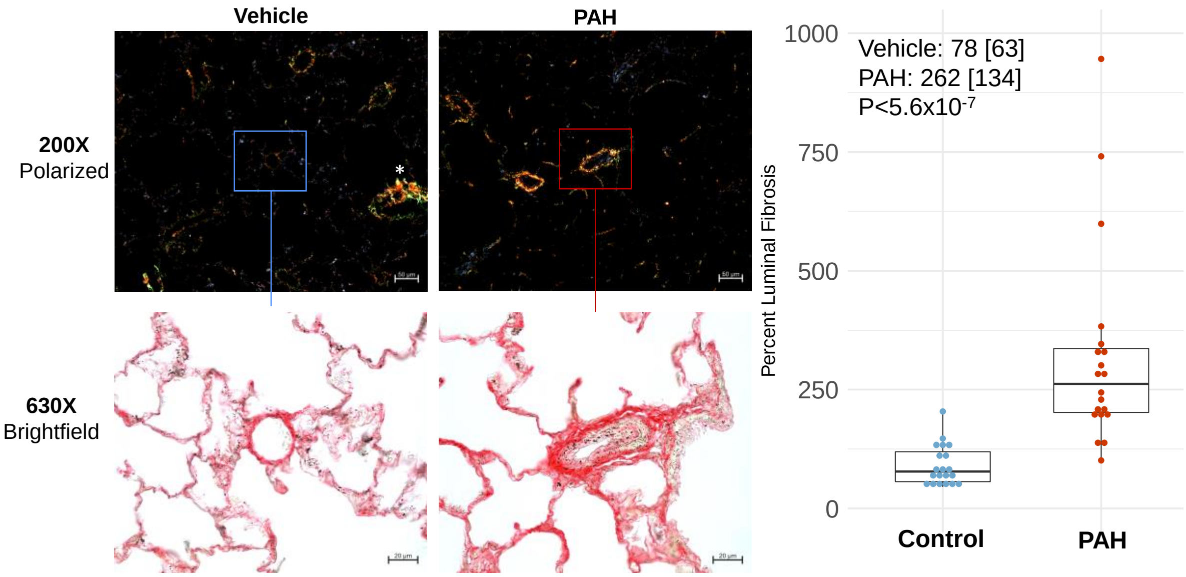

Supplemental Figure 11. Inflammatory pulmonary arterial hypertension (PAH) in vivo is associated with fibrotic remodeling of pulmonary arterioles. Sprague Dawley rats were administered a single intraperitoneal injection of monocrotaline (MCT) 50 mg/kg) or vehicle control (V) and lung tissue was harvested 23-25 days later. Formalin-fixed paraffin-embedded lung tissue sections were stained with picrosirius red, to emphasize fibrillar collagen, and were analyzed by brightfield (630X, inset, scale bar = 20μm) and polarized light microscopy (200X, scale bar = 50μm). Collagen was quantified in pulmonary arteriolar vessels in brightfield images using ImageJ (collagen area in vessel wall/luminal area) x 100. Asterisk corresponds to an airway and serves as a positive control for the presence of lung collagen. Median [IQR], 5 representative vessels matching caliber and remodeling pattern of vessels used in MIMS analysis/rat, 4 rats/condition. P-values calculated by the Mann-Whitney test.

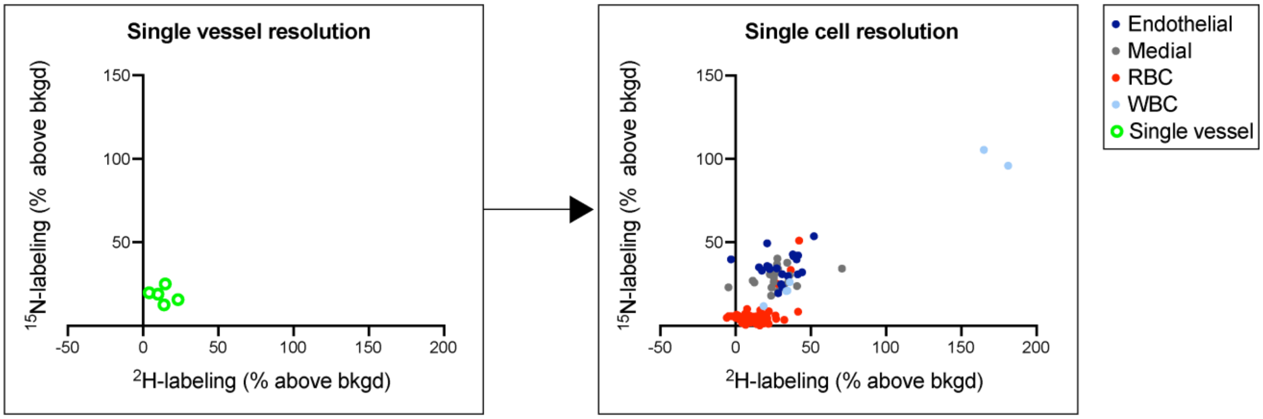

Supplemental Figure 12. Single cell resolution label quantification reveals heterogeneity of metabolism. Data from a representative animal with MCT PAH demonstrates how single cell resolution measurements capture heterogeneity within and between cell types that is not appreciated with vessel-level measurements. For the vessel level analyses on the left, regions of interest (ROI) were applied to the vessel inclusive of mural cells, endothelial cells, and the intraluminal blood cells. On the right, ROI were applied to individual cells for the corresponding vessels captured at vessel-scale (left). Abbreviations: bkgd, background; MCT, monocrotaline; RBC, red blood cell; WBC, white blood cell.

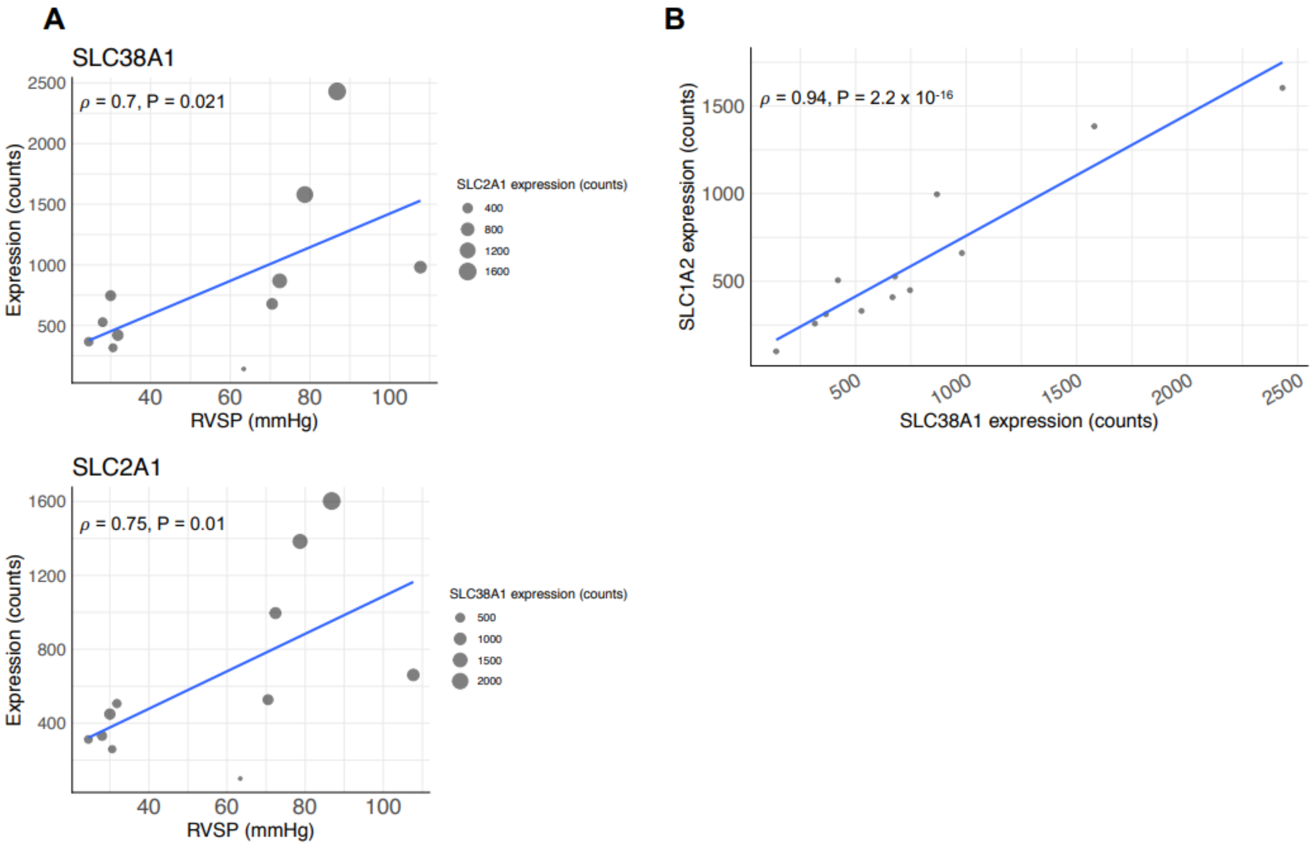

Supplemental Figure 13. Proline and glucose transporter expression in pulmonary artery endothelial cells are functionally associated with pulmonary hypertension in vivo. Right heart catheterization was performed In MCT and vehicle-treated rats undergoing PAEC isolation (N=6/condition, day 23-25 after MCT injection) using a fluid-filled catheter and expression of proline and glucose transporters was determined by RNA sequencing and expressed as gene counts. RVSP could not be obtained in one animal. **(A)** A significant correlation was identified between RVSP, a marker of pulmonary hypertension severity, and expression of SLC38A1 (a transporter of proline and other amino acids) and SLC2A1 (a transporter of glucose, also known as GLUT1). **(B)** Expression of SLC38A1 and SLC1A2 is strongly correlated in rat PAECs, emphasizing the potential for anabolic convergence between glucose and proline in inflammatory PAH. Abbreviations: MCT, monocrotaline; PAEC, pulmonary artery endothelial cell; ρ, Spearman's correlation coefficient; RVSP, right ventricular systolic pressure; SLC38A1, solute carrier family 38A1, SLC2A1, solute carrier family 2A1.
